# Sequencing refractory regions in bird genomes are hotspots for accelerated protein evolution

**DOI:** 10.1186/s12862-021-01905-7

**Published:** 2021-09-18

**Authors:** R. Huttener, L. Thorrez, T. In’t Veld, M. Granvik, L. Van Lommel, E. Waelkens, R. Derua, K. Lemaire, L. Goyvaerts, S. De Coster, J. Buyse, F. Schuit

**Affiliations:** 1 Gene Expression Unit, Department of Cellular and Molecular Medicine, KU Leuven, Herestraat 49, O&N1, bus 901, 3000 Leuven, Belgium; 2 Tissue Engineering Laboratory, Department of Development and Regeneration, KU Leuven Campus Kulak, Kortrijk, Belgium; 3 Laboratory of Protein Phosphorylation and Proteomics, KU Leuven, Leuven, Belgium; 4 Laboratory of Livestock Physiology, Department of Biosystems, KU Leuven, Leuven, Belgium

**Keywords:** Avian genomes, Evolution, Accelerated, Sequencing artifacts, Transcript landscapes, Missing genes, GLUT4, *SLC2A4*, *ENO3*, *ALDOA*, *PYGM*

## Abstract

**Background:**

Approximately 1000 protein encoding genes common for vertebrates are still unannotated in avian genomes. Are these genes evolutionary lost or are they not yet found for technical reasons? Using genome landscapes as a tool to visualize large-scale regional effects of genome evolution, we reexamined this question.

**Results:**

On basis of gene annotation in non-avian vertebrate genomes, we established a list of 15,135 common vertebrate genes. Of these, 1026 were not found in any of eight examined bird genomes. Visualizing regional genome effects by our sliding window approach showed that the majority of these "missing" genes can be clustered to 14 regions of the human reference genome. In these clusters, an additional 1517 genes (often gene fragments) were underrepresented in bird genomes. The clusters of “missing” genes coincided with regions of very high GC content, particularly in avian genomes, making them “hidden” because of incomplete sequencing. Moreover, proteins encoded by genes in these sequencing refractory regions showed signs of accelerated protein evolution. As a proof of principle for this idea we experimentally characterized the mRNA and protein products of four "hidden" bird genes that are crucial for energy homeostasis in skeletal muscle: *ALDOA*, *ENO3*, *PYGM* and *SLC2A4*.

**Conclusions:**

A least part of the “missing” genes in bird genomes can be attributed to an artifact caused by the difficulty to sequence regions with extreme GC% (“hidden” genes). Biologically, these “hidden” genes are of interest as they encode proteins that evolve more rapidly than the genome wide average. Finally we show that four of these “hidden” genes encode key proteins for energy metabolism in flight muscle.

**Supplementary Information:**

The online version contains supplementary material available at 10.1186/s12862-021-01905-7.

## Background

The enigmatic evolutionary history of birds has tantalized investigators since more than a century [1, 2]. A major paradigm shift was the idea that all lineages of modern birds descended from a line of theropod dinosaurs that survived the fifth mass extinction event 66 million years ago [2–4]. This new paradigm makes one wonder about evolutionary steps that caused a lineage of large predatory theropod dinosaurs to diverge into a lineage comprising a two gram weighing nectar drinking hummingbird. The interest in this evolutionary enigma is high for several reasons. One avian species, *Gallus gallus*—the domesticated chicken—has been a model organism in biomedical research for more than a century [5]. The chicken genome was one of the first sequenced non-mammalian vertebrate genomes [6], and has since then attracted a large research community. In addition, poultry research has widespread economic importance as chicken is one of the most used animals for human nutrition [7]. Despite the active chicken genome consortium and massive sequencing projects involving large sets of avian genomes [3, 8, 9], an inexplicable large number of avian genes remains unaccounted for [3, 10]. Curiously, many of these genes have orthologs in fish species, other reptilian lineages and in mammals. For instance Howe et al. (2013) showed that the chicken genome is missing 2059 genes which are present in the human, mice and zebrafish genomes [11]. This finding was confirmed by Lovell et al. (2014) who found 1559 genes missing in chicken and zebra finch but present in non-avian vertebrates [10]. When the search for missing avian genes was extended by the study of 60 different bird genomes, half of the genes could be found in at least one of the other bird genomes [10]. It was suggested that a massive gene loss may have been partially compensated by paralogous genes [3, 10]. One third of the “missing” genes were found to be regionally clustered in non-avian sauropsid genomes and the human genome [10]. Hron et al. (2015) later showed that a small fraction of those genes, presumed to be lost in birds, could actually be retrieved in raw RNAseq data [12]. These so called "hidden" genes had a high GC content, suggesting that a fraction of previously reported missing genes was not lost during evolution. Commonly used sequencing technologies are hindered by GC bias [13, 14], impacting the sequencing and annotation process. Resequencing the chicken genome with a modified method that generated longer reads, resulted in the identification of 121 new genes [15]. Botero-Castro et al. (2017) recovered between 519 and 1775 “missing” genes in several avian species [16]. These recovered genes had a substantially higher GC content than previously annotated genes [16]. Using a de novo approach, Yin et al. (2019) also indicated that a large amount of avian “missing” genes are an artifact caused by high GC content [11, 17].

The interest in the phenomenon of "hidden" genes is further sparked by the fact that some of these genes encode crucial mediators or regulators of vertebrate physiology [11, 17]. One example is the leptin gene which encodes a satiety-inducing hormone produced by adipocytes [18, 19]. Interestingly, the avian leptin gene was unaccounted for a decade and only deciphered after painstaking sequencing of DNA with very high GC content [20–22]. Another example is the *SLC2A4* gene, which encodes the insulin-regulated glucose transporter GLUT4 [23]. Curiously, in contrast to other vertebrates neither *SLC2A4* mRNA nor GLUT4 protein were detected in birds and this absence was causally connected to the fact that birds have severe insulin resistance and relatively high blood glucose levels when compared to human standards [24, 25].

Together, intensive research efforts by many laboratories have not solved the mystery of more than 1000 common vertebrate genes that are currently annotated as missing in most if not all sequenced bird genomes. Such a large number of genes, often encoding key mediators or regulators of function, seems problematic in biological terms and seems far too high in terms of gene loss during the evolution of the avian lineage. In the present work, we have approached the enigma of the "missing/hidden" bird genes by genome landscapes in which a sliding window analysis smoothens the erratic behavior of individual genes so that typical regional effects are visualized in different parts of a vertebrate genome [26]. The technical hindrance of gene annotation by regions with high GC content predicts that also in other genomes “hidden” genes may be clustered in specific areas. We therefore also examined the concurrence of “missing genes” and high regional GC content in non-avian taxa.

## Results

### Genome landscapes visualize clusters of "missing" bird genes

To search for regional effects of "missing" bird genes we undertook a comparative analysis of eight avian genomes we selected on basis of representation of the major avian clades and best annotated in terms of number of protein encoded genes. The avian genomes were compared to the genomes of alligator, turtle, lizard and snake (*Alligator mississippiensis*, *Chrysemys picta bellii*, *Pogona vitticeps*, *Python bivittatus*) and two reference genomes (*Homo sapiens* and *Lepisosteus oculatus*). A list of 15,135 vertebrate protein encoding genes was assembled based on annotation in the human genome as well as in in at least one non-avian vertebrate (Additional file 1: Table S1). From this list 1026 genes were not found in any of the eight examined bird genomes. For each gene, the presence index (PI) was calculated: the fraction of bird genomes in which the gene is annotated:

$${\text{PI}}^{{{\text{geneX}}}} = {{\text{number of avian genomes in which gene X is annotated}} \mathord{\left/ {\vphantom {{\text{number of avian genomes in which gene X is annotated}} 8}} \right. \kern-\nulldelimiterspace} 8}$$

**Fig. 1:**
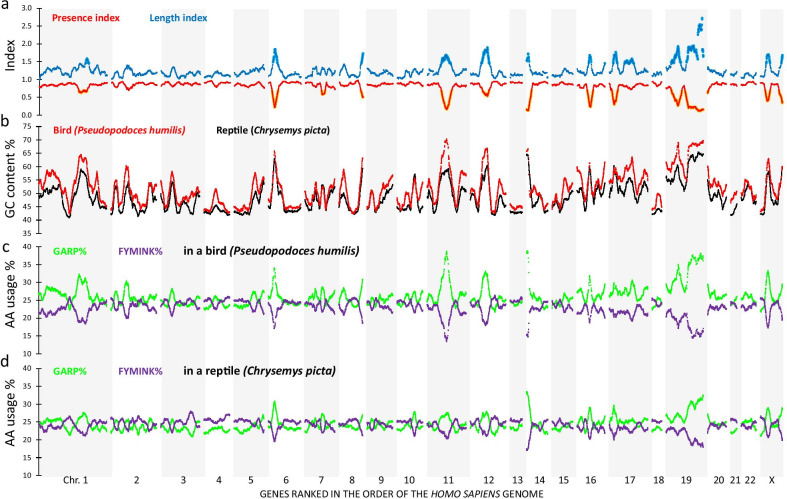
Avian and non-avian reptilian landscapes of protein encoding genes. A set of 15,135 common vertebrate genes was sorted in the order of the human reference genome, alternating grey/white bars represent the different chromosomes. A sliding window of a centered gene and its 100 neighbors was taken to calculate the regional genomic average for each variable. **a** Presence index (red) and length index (blue) of the genes in the eight avian genomes. The areas in orange dots define the genes where the presence index is below the threshold of 0.70. In light blue it is shown where the length index is higher than the threshold of 1.46. In panel **b**, we have displayed the GC content of mRNA transcripts of the best annotated of the 4 studied non-avian reptiles (black, *Chrysemys picta*) and eight studied birds (red, *Pseudopodoces humilis*). The highest peaks of GC content are often seen in areas of a low presence index. **c** and** d** Landscapes of the cumulative presence of GARP% (encoded by GC-rich codons, green) or FYMINK% (encoded by AU-rich codons, purple) in the *Pseudopodoces humilis* (**c**) and *Chrysemys picta* (**d**) genome. The amount of GARP% and GC content are strongly correlated (R = 0.92 for *Chrysemys picta* and R = 0.91 for *Pseudopodoces humilis*)

This index can theoretically vary between 0 (gene present in none of the bird genomes) to 1 (gene present in all 8 bird genomes). Figure 1a (red line) shows the regional mean of this index using a sliding window containing the averaged value of a gene with its 50 neighbors on both sides:

$$^{{{\text{SW}}}} {\text{PI}}^{{{\text{geneX}}}} = {\text{Mean of all PI measurements between position PI}}^{{{\text{geneX}} - {5}0}} {\text{and position PI}}^{{{\text{geneX}} + {5}0}}$$

Using the human reference genome as a basis to assess regional effects, it can be seen that the presence index fluctuates between baseline value of approximately 0.86 and regions where values drop to much lower levels, approaching 0.1 in some areas. The distribution of the 15,135 sliding window values showed a median value of 0.86, but a wide left tail with low index values can be seen (Additional file 2: Fig. S1a). As a control, we scrambled the position of the 15,135 genes and generated 1000 random sets of 101 genes to calculate scrambled presence index values (compare the red window distribution to the grey random distribution in Additional file 2: Fig. S1a). With assumption of random sampling with replacement, the expected number of values under 0.7 is 30. However, the observed number in the genome landscape is 2187 (P ≪ 0.001). Such large number of windows supports the idea of massive amounts of "missing" avian genes as was previously described in the literature [3, 6, 10–12, 27, 28]. From the genes located in these windows, 613 were not found in any bird genome. Interestingly, the windows with presence index values below the threshold value of 0.70 were clustered in 14 distinct regions on the human genome (highlighted in yellow in Fig. 1a). To exclude the scenario in which existing orthologues were missed due to the matching algorithm (e.g. by a mismatch of gene names such as LOCnumber instead of common gene symbol), we manually analyzed each of the genomes for “missing genes” that were indeed present under a different name (LOCnumber) using gene description, sequence homology and synteny as criteria. Such labor-intensive manual analysis of all analysed genomes, resulted in an extra 9013 genes (on average 751 per genome) that could be added to the database. This resulted in a dataset of 15,624 genes which were present in the human genome and at least one non-avian reptile. However, these manual additions did not fundamentally alter the presence index plots (Additional file 3: Fig. S2a, b). Indeed, the same 14 regions of the human genome encode clustered genes that are massively "missing" in bird genomes. With fully automated annotation 6.8% of the common vertebrate set was "missing" (1,026/15,135); after manual additions this was lowered to 5.3% (833/15,624). It can also be argued that the analysis of only eight avian genomes was responsible for a relatively high number of “missing genes” and that the inclusion of additional bird genomes can be expected to change the "presence index" reducing the depth of the valleys in the 14 hot spots. We therefore re-analyzed the clustering of “missing genes” in a much larger set of avian genomes (75 instead of 8, using a new calculation of PI^geneX^ = number of avian genomes in which the gene is annotated/75). The results show that the clustering of “missing genes” remains essentially unaltered by the extension of analysis to the large majority of currently sequenced bird genomes (Additional file 3: Fig. S2c).

**Fig. 2:**
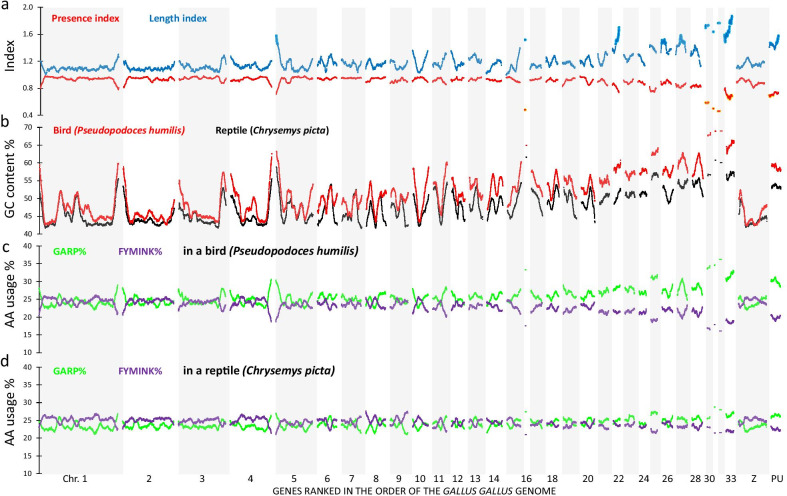
Avian and non-avian reptilian landscapes of protein encoding genes in the chicken genome order. The same data as in Fig. 1 are shown, but now the genes are ranked in the order of the chicken genome. For 214 genes, chicken chromosomal position is unknown (PU). **a** presence and length indices for birds indicate that most gene information (number of genes and sequence) is missing in the microchromosomes. **b** The GC content of *Pseudopodoces humilis* is the highest at the subtelomeres of the macrochromosomes and in the microchromosomes. Note that in the macrochromosomes the GC content in both species is more similar than in the microchromosomes. **c** and **d** GARP% and FYMINK% of the predicted proteins in *Pseudopodoces humilis* and *Chrysemys picta*

One could argue that the human reference genome is not the right choice to visualize regional effects of "missing" avian genes. We addressed this point by recalculating the landscapes of presence index using the gene order of two other vertebrates: the spotted gar (*Lepisosteus oculatus*) and chicken. The first is a bony fish that did not evolve with a teleost genome duplication event; moreover, this genome is well annotated and most of the genes are mapped to its 28 chromosomes. Also in the presence index landscape of the *Lepisosteus oculatus* genome order, the "missing" genes of avian genomes are strongly clustered to specific regions of the fish genome, particularly subtelomeres of the large chromosomes 1 and 2 and the small chromosomes 24 and 28 (Additional file 4: Fig. S3a). We also calculated the presence index using the chicken reference genome, whereby all genes (2633) that are not listed as present in this genome were excluded (Fig. 2a). This analysis therefore searches for genes that are present in chicken but absent in other avian genomes. Despite this limitation, this landscape clustered the missing genes primarily to the microchromosomes (e.g. CHR30 and CHR33). For the macrochromosomes, only small deviations from the maximal score of 1.0 are seen in subtelomeric regions. A low presence index is also associated with genes that are listed as present (sequence available) in the chicken genome database but not mapped to the chicken reference genome (214 genes position unknown (PU), see Fig. 2a).

### Avian transcripts in the clusters of "missing" bird genes are often predicted from gene fragments

The enrichment of "missing" bird genes in microchromosomes and in the group that is not yet mapped on the chicken reference genome, points in the direction of technological hindrance rather than a biological explanation for the “absence” of genes from annotated genomes. Hindrance in the completeness of sequencing was also noticed when comparing the relative length of all predicted mRNA transcripts in the clusters of missing genes and genome loci away from these clusters. This was analyzed by calculating the length index (LI):

$${\text{LI}}^{{{\text{geneX}}}} = {{\text{mean transcript length of X in the non - avian genomes}} \mathord{\left/ {\vphantom {{\text{mean transcript length of X in the non - avian genomes}} {\text{mean avian transcript length of X}}}} \right. \kern-\nulldelimiterspace} {\text{mean avian transcript length of X}}}$$

Also for LI a regional average was calculated via a sliding window encompassing gene X and its 100 neighbors:

$$^{{{\text{SW}}}} {\text{LI}}^{{{\text{geneX}}}} = {\text{Mean of all LI measurements between position LI}}^{{{\text{geneX}} - {5}0}} \;{\text{and position LI}}^{{{\text{geneX}} + {5}0}}$$

A high length index means that on average the transcripts are shorter in birds than in the other vertebrates. The result is a genome-wide landscape with a shape that strikingly mirrors the presence index. Indeed the blue lines for the human reference genome (Fig. 1a), the *Lepisosteus oculatus* reference genome (Additional file 4: Fig. S3a) or the chicken reference genome (Fig. 2a) mirror the red lines of the presence index (correlation coefficient R = − 0.85). The key element of this symmetry is that the average avian transcript length is reduced compared to non-avian orthologues selectively in the sites were avian genes are "missing;. This outcome makes genome compactness of avian genomes an unlikely explanation, as this would elevate the baseline of the length index on a genome wide basis rather than causing discrete maxima. The interpretation is that avian genes annotated within the “missing” gene clusters have been sequenced incompletely. To ascertain the non-randomness of this phenomenon, we constructed 1000 random sets of 101 genes to calculate length indices after scrambling gene positions (Additional file 2: Fig. S1b). The distribution of these randomized sets (grey) was fundamentally different from the distribution of the length index values using the human reference genome (blue). The highest length index value found from this random set was 1.46 (P < 0.001 to obtain a length index > 1.45). When we then applied the value of 1.46 to the landscape of ordered genes we obtained 1214 windows that exceeded this threshold (P ≪ 0.001). Of these 1214 windows, 973 were common to the windows with a lower presence index than the 0.1% threshold (Additional file 2: Fig. S1c).

### Clusters of "missing" bird genes are characterized by high GC content and altered codon usage

It is known that GC-rich sequences can pose technical problems in the sequencing and annotation pipeline [13, 14, 29], these sequences have therefore also been described as sequencing refractory DNA [30]. We assessed the GC-content of the best annotated bird genome: *Pseudopodoces humilis* (Fig. 1b, red). This was compared to the genome of *Chrysemys picta* (Fig. 1b, black) which contains 1287 more annotated genes from the common gene set than the chicken genome. In the clusters of “missing” genes, the expected scarcity of data is clearly visible, yet the nature of the rise in GC content is well conserved. Interestingly, and consistent with a technical sequencing artifact, the genome-wide distribution of transcript GC content peaks (often above 60%) in clusters coinciding exactly with the regions where the avian presence index presented minimal values while the length index peaked (Fig. 1a versus Fig. 1b). The Pearson correlation between length index and GC% is very strong (R = 0.805), as well as the correlation between presence index and GC% (R = − 0.704). In other words: regions where GC% in the *Pseudopodoces humilis* genome increased to 60% or more coincided with regions with clustered lack of parts of the predicted mRNA sequence in the avian genes. Therefore, the genome-wide landscape analysis of presence and length indices indicates that sequencing refractory DNA rather than evolutionary loss may explain the "missing" gene information in avian genomes. When the genes were ordered on basis of the chicken genome (Fig. 2b) the landscapes of GC% of *Pseudopodoces humilis* displayed peaks mainly at the subtelomeres for the macrochromosomes (Chr1–5, ChrZ) and an increase over landscapes of *Chrysemys picta* for most of the microchromosomes (Chr6–Chr33). Of interest is the fact that for six chicken microchromosomes (chr.29, 34–38) not a single gene has been mapped until now.

The relative occurrence of glycine (G), alanine (A), arginine (R) and proline (P) in the amino acid composition of proteins (GARP%) represents amino acids encoded by codons of which the first two codon bases are either guanine or cytosine. On the contrary, the relative occurrence of phenylalanine (F), tyrosine (Y), methionine (M), isoleucine (I), glutamine (N) and lysine (K) (FYMINK%) represents residues encoded by codons of which the first two codon bases are adenosine or uracil. We have shown before in vertebrate genome landscapes [26] that the landscapes of GARP% and FYMINK% are strongly negatively correlated and that GARP% closely matches the landscape of GC%. In the present study, we observed that the landscapes of GARP% (green lines) and FYMINK% (purple lines) both in a bird (*Pseudopodoces humilis—*Fig. 1c) and in a turtle (*Chrysemys picta—*Fig. 1d) deviate from the baseline value of approximately 25% in regions where GC% rises. As expected from our previous work [26] GARP% and GC% are nearly perfectly positively correlated (R > 0.91) while a strong negative correlation is seen between FYMINK% and GC% (R < − 0.95). When plotted in the gene order of the chicken genome the GARP% and FYMINK% were similar over most of the *Chrysemys picta* genome, whereas for of *Pseudopodoces humilis* over most parts of the microchromosomes GARP% was clearly higher than FYMINK% (Fig. 2c and d).

### Heatmap representation of landscapes of GC content and protein divergence rates

**Fig. 3:**
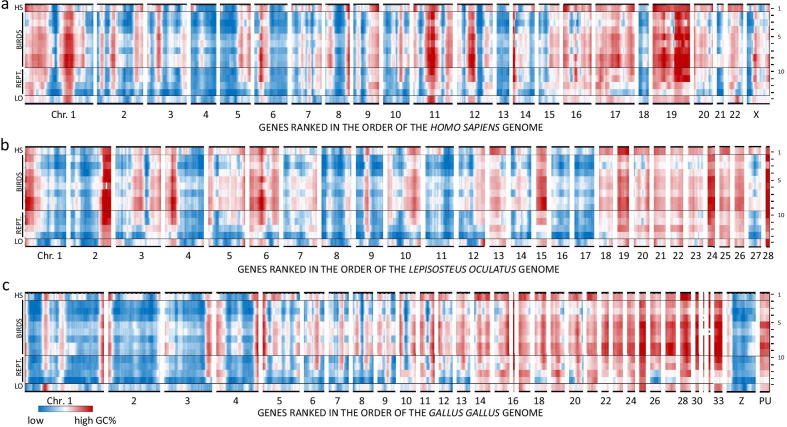
Heatmap of GC content profiles. The GC content of the predicted mRNA transcripts of eight birds, and four non-avian reptiles is shown together with the two reference genomes (*Homo sapiens* (HS) and *Lepisosteus oculatus* (LO)) using a heatmap display. Genes were positioned according to the order of the human genome **a**), the *Lepisosteus oculatus* genome (**b**) or the *Gallus gallus* genome (**c**). Most intense red (highest GC%) is found in the avian genomes (lines 2–9), typically in microchromosomes or subtelomeric in macrochromosomes when genes were ranked according to the chicken genome. When genes were ranked according to the human or gar genome, many regional GC maxima for birds were located far from the chromosomal ends. Numbering: 1 *Homo sapiens*, 2 *Apteryx australis*, 3 *Struthio camelus*, 4 *Anser cygnoides*, 5 *Gallus gallus*, 6 *Calypte anna*, 7 *Aquila chrysaetos*, 8 *Pseudopodoces humilis*, 9 *Sturnus vulgaris*, 10 *Alligator mississippiensis*, 11 *Chrysemys picta*, 12 *Pogona vitticeps*, 13 *Python bivittatus*, 14 *Lepisosteus oculatus*

**Fig. 4:**
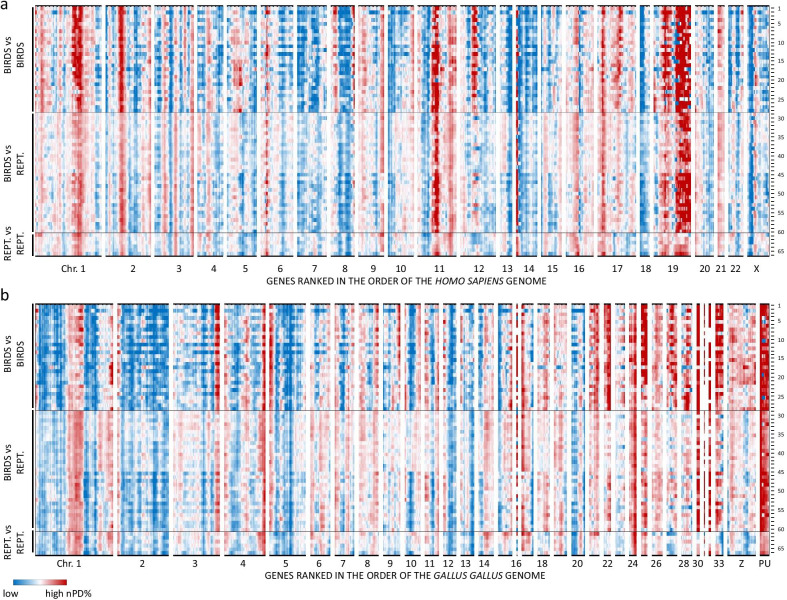
Heatmaps of normalized protein divergence (nPD%). For each pair of orthologous proteins of two species the measured % of divergence (100—%identity) was normalized by the genome-wide average of % divergence. A sliding window of 101 genes generates data that can highlight regions where proteins diverge faster (red) or slower (blue) than the genome wide average. Heatmaps were made with the gene order of the human genome (**a**) and chicken genome (**b**). Note typically high rates of protein divergence in the chicken microchromosomes and in genes where mapping in the chicken genome is still unknown (PU). Individual lines represent three different groups of comparisons: avian//avian (1–28), avian//non-avian reptile (29–60), non-avian reptile//non-avian reptile (61–66)

In Figs. 1b and 2b, we compared landscapes between two genomes only. To assess a phylogenetic basis for the landscape details, we plotted GC% landscapes of all 14 studied genomes in a heatmap display, using the gene order of *Homo sapiens* (Fig. 3a, mammal), *Lepisosteus oculatus* (Fig. 3b, fish) and *Gallus gallus* (Fig. 3c, bird). Irrespective of the chosen reference genome, bird genomes were observed to contain regions with the highest GC%. Moreover, when the genes were ordered according to the chicken reference genome (Fig. 3c) the areas of high GC% were more likely in microchromosomes, while in macrochromosomes, GC enrichment was preferentially observed in subtelomere regions. Together, these landscapes show a clear regional effect on the amino acid composition of proteins which is the result of a regional effect of the base composition of encoded mRNA. The next question was whether a regional effect on amino acid composition could have an effect on the rate of protein divergence. We next calculated normalized protein divergence profiles, which show protein divergence—as a measure of protein evolution—in function of the corresponding gene position on the genome. Protein divergence was calculated for each pair of orthologous proteins and normalized for the genome averaged divergence rate. The resulting normalized protein divergence (nPD%) was calculated for all comparisons within the group of birds (8 species; 28 pairwise comparisons) and for the non-avian genomes (4 species; 6 pairwise comparisons) as well as for the comparisons between an avian and non-avian species (32 comparisons—a list of the pairwise comparisons between any two species in this study is provided in Additional file 5: Table S2). The overall outcome of this analysis is shown as a heatmap (Fig. 4), either in the gene order of the human reference genome (Fig. 4a) or of the chicken genome (Fig. 4b). Using this graphical representation it can be clearly seen that regions with highest regional GC% levels (Fig. 3) coincide with regions that have the highest nPD% for intra-avian comparisons (lines 1–28 in Fig. 4). Moreover, when ordered for the chicken reference genome, the highest nPD% was observed in some of the microchromosomes and at the subtelomeres of macrochromosomes. The 214 genes that are listed in the chicken reference genome as "position unknown" (PU) have uniformly high GC% (Fig. 3) and nPD% (Fig. 4). Together, these results strongly indicate that precisely in the regions with “hidden” genes, where GC% and GARP% are high, the average rates of protein evolution has increased over a macro-evolutionary time frame as compared to regions with low GC%, low GARP% where “missing” genes are less common.

### Proof of principle of the expression of "hidden" genes

Many "missing" bird genes cluster in DNA regions where elevated GC% is an obstacle for sequencing. Moreover, accelerated divergence of encoded proteins in these regions is a challenge for correct annotation. This may explain the clustering of “missing” genes in these regions with the prediction that such genes “hidden” by the circumstances mentioned above wait to be discovered. This seems an important idea in order to better understand avian biology as many of the "missing" genes encode proteins which are known in other vertebrates to play crucial roles in energy homeostasis. As a proof of principle that some of the “missing” genes are “hidden” by the high GC%, we elucidated the full length coding sequence and expression of four poorly characterized avian genes that are pivotal for glycolytic ATP production in vertebrate fast type II muscle fibers: the muscle type glycolytic enzymes aldolase A (*ALDOA*) and enolase 3 (*ENO3*), the muscle type glucose transporter GLUT4 (*SLC2A4*) and the muscle type glycogen phosphorylase (*PYGM*). Prior to our analysis, we could only find two (partial) sequences of *ALDOA* and two (partial) sequences of *PYGM*. For *ENO3*, no annotations were found in our set of 8 bird genomes but it was subsequently found in the larger set of bird genomes (5 out of 75). In contrast, for *SLC2A4*, no bird sequences were found. Starting from chicken pectoralis muscle mRNA and a partial sequence from gene fragments present in one or more avian genome databases, nested primers were used to complete the full length predicted mRNA sequence. A more detailed description of the cloning steps for each gene can be found in Additional file 6 (description, used gene fragments from different species, used primers and the identified sequences). We identified the full-length mRNA sequence and thereby demonstrated that these genes are not “missing”, but have thus far been “hidden”. BLAST of the four sequences against the human reference genome confirmed the human paralogous gene as the best match. *ALDOA, ENO3, PYGM* and *SLC2A4* were indeed located in the hereabove identified “missing” gene clusters on human chromosome 16, 17, 11 and 17 respectively. All four transcripts had high average GC content (respectively, 65%, 62%, 62% and 69%). As expected from the base composition, the GARP% in the four proteins was higher than the genome wide average of 25% (respectively 30%, 28%, 27%, and 40%).

**Fig. 5:**
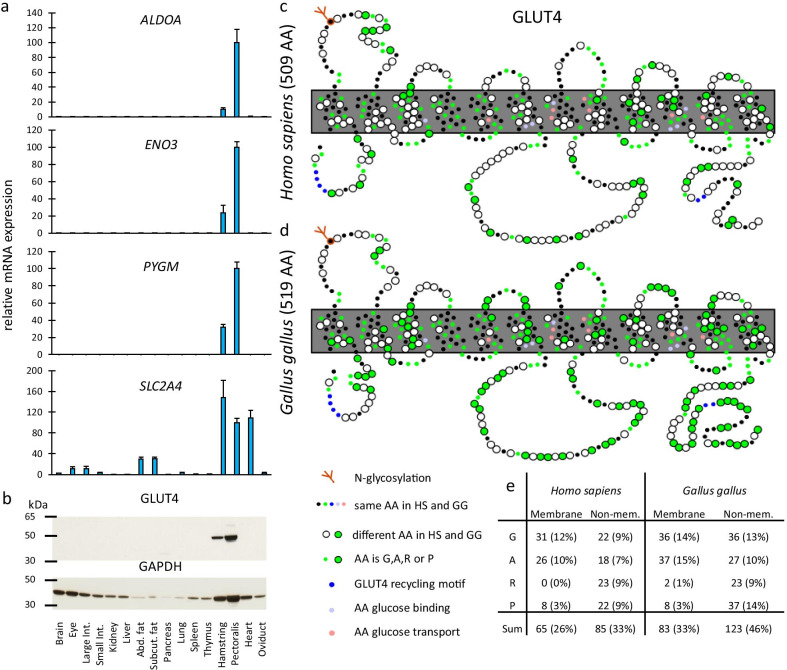
Proof of concept of four "hidden" chicken genes. **a** After obtaining the complete coding sequence we assessed by quantitative PCR the expression profiles of muscle-type aldolase (*ALDOA*), enolase (*ENO3*), glycogen phosphorylase (*PYGM*), and glucose transporter (GLUT4). Expression signals were normalized against ribosomal protein gene *RPS13* [74] and calculated relative to the expression ratio in pectoralis muscle. **b** Western blot of immunoreactive GLUT4 using protein extracts from the same tissue panel as for the mRNA analysis. Lower panel shows abundance of glyceraldehyde-3-phosphate dehydrogenase (GAPDH). **c** and **d** Schematic representation of human (**c**) and chicken (**d**) GLUT4 primary structure in a model of 12 transmembrane helices that surround the water-filled glucose diffusion pore [23]. Small circles, identical residues in both species; green, one of the following four amino acids (GARP) encoded by GC-rich codons. Residues that are important for sugar binding and transport and for GLUT4 recycling are conserved (violet and pink circles). **e** Counts (%) of glycine, alanine, arginine and proline in membrane and non-membrane parts in the human and chicken GLUT4. The avian increase in GARP is not random. For instance, the number of helix-disrupting prolines only rise in the non-membrane segments of the protein

Next, the mRNA expression in 16 different chicken tissues was examined by quantitative PCR and the highest expression signals for each of the four genes were found in skeletal muscle (Fig. 5a). Given the currently unresolved question whether or not chicken glucose homeostasis is influenced by an insulin regulated glucose transporter in skeletal muscle [25] we compared in more detail the evolution of chicken GLUT4. Of interest is that the divergence of chicken versus human GLUT4 protein (42%) is far greater than alligator versus human GLUT4 (32%) or turtle versus human GLUT4 (27%). For the details of protein evolution we used the accepted structural model based on 12 transmembrane helices that surround a water filled glucose diffusion pore [23]. As shown in Fig. 5c, d, the predicted chicken GLUT4 has a well-preserved structure including an *N*-glycosylation site in the first luminal/extracellular loop and conserved residues for sugar binding and transport. Furthermore, both the FQQI and LL-motifs which are unique for GLUT4 and functionally required for the recycling pathway of GLUT4 [31–34], are conserved in the chicken structure (blue circles in Fig. 5c, d). On the contrary, residues distal to the LL-motif close to the C-terminus, which in mammals are associated with insulin regulation [32, 34] are less conserved in the chicken sequence (Fig. 5c, d). Furthermore, in line with increased GC% and GARP% in the "missing" gene clusters, the number of GARP residues encoded by GC-rich codons increased from 150 in human GLUT4 to 206 in chicken GLUT4 (green circles in Fig. 5c–e). Although a relative increase in GC% may entail a rise of GARP%, the distribution of glycine/alanine (allowed in transmembrane helices) and arginine/proline (avoided in transmembrane helices) is not random (Fig. 5e).

We collected additional evidence for expression of these "hidden” genes at the protein level. Different peptides from ALDOA, ENO3 and PYGM could be detected in total protein extracted from the cytosol fraction of chicken pectoralis muscle using data dependent mass spectrometric analysis (posterior error probability < 0.01). Chicken GLUT4 peptides could not be detected in these extracts. However, using a polyclonal rabbit anti-chicken GLUT4 antibody, immunoblotting revealed an immunoreactive protein in skeletal muscle (Fig. 5b). Moreover, after immunoprecipitation of GLUT4 from total chicken pectoralis muscle lysate using the same antiserum, we could detect peptides from the predicted chicken GLUT4 sequence using data dependent mass spectrometry (posterior error probability < 0.01). A list of the detected peptides for the different proteins is given in Additional file 7: Table S3. One of these peptides covers the C-terminus of GLUT4 showing an altered insulin regulation motif, confirming the correctness of the predicted protein sequence from the sequenced muscle mRNA.

### Proof of principle of clustered "hidden genes" in GC rich areas of non-avian vertebrate genomes

**Fig. 6:**
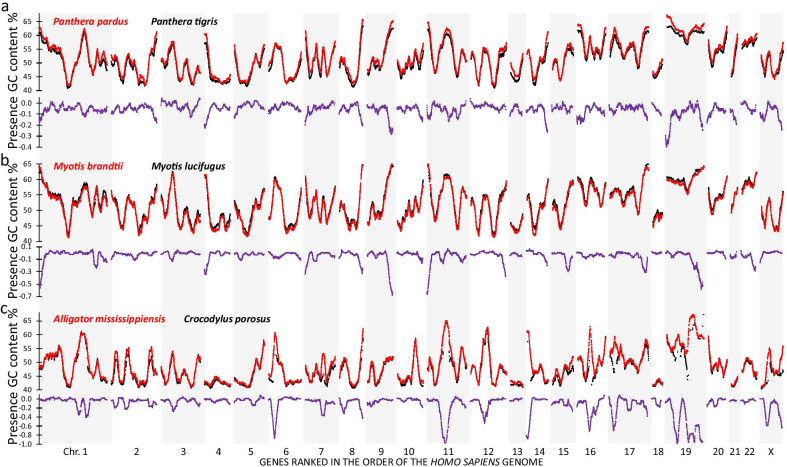
Effect of GC content on “missing genes” in non-avian genomes. For each three genome comparisons (Panthera, **a**; Myotis, **b**; Crocodylia, **c**), the upper panel shows the GC content landscapes of the two species. In red, the species with the highest number of genes in the comparison is shown, while the less well annotated genome is shown in black. The lower panels represent the presence score, which was calculated for each gene as follows: − 1 = only present in the best annotated genome; 0 = present in both genomes; + 1 = only present in the least annotated genome. A sliding window of 101 genes was applied to the presence score and GC% showing a clearcut correlation between the two parameters. Thus, also in non-avian genomes a higher number of "missing" genes is found in regions with elevated GC%

The present study adds to a growing mass of evidence that the massive amount of “missing” genes in avian genomes reflect the lagging of sequencing and annotation information in particular for GC% rich. There is no reason to assume that this relationship between high GC% and sequencing refractoriness is specific to birds. Therefore, we assessed this relationship in three non-avian genome pairs, in which one species was notably more completely sequenced and annotated than the other (Fig. 6). For instance, when comparing the genomes of two closely related panthers (*P. pardus* and *P. tigris*), the former is far more complete (855 extra genes) than the latter. We calculated a presence score by giving a score to each gene: − 1 if the gene is not found in the less complete genome, 0 if the gene is found in both species and + 1 if the gene is only present in the less complete genome. On the presence score, we applied the sliding window, making the average of its 100 neighbors. As shown in Fig. 6a the "missing" *P. tigris* genes cluster in subtelomeric regions (aligned to the human reference genome), precisely in regions where the GC% of predicted mRNAs rises. A similar result is obtained when comparing the genomes of two Myotis bat species: *M. brandtii* and *M. lucifugus*. *M. lucifugus* is "missing" 569 genes compared to *M. brandtii*. Further generalization was confirmed by the comparison of two crocodile genomes: the more complete *Alligator mississippiensis* and the less complete *Crocodylus porosus* (1480 genes less annotated). While in the human reference genomes the areas of "missing" genes are often non-telomeric (Fig. 6c), the landscapes of the crocodile genomes with the chicken reference genome (Additional file 8: Fig. S4) shows enrichment of “missing” genes in subtelomeres of macrochromosomes and entire microchromosomes. These data illustrate that the phenomenon of clustering of “missing” genes GC% rich genome areas is not specifically present in bird genomes but also present in the genomes of other vertebrates.

## Discussion

The publication of the first draft of the chicken genome already mentioned the absence of a surprisingly large number of genes that were present in other vertebrate genomes [6]. This initiated a debate whether or not this absence represented a true loss of genes in the avian lineage or an artifact created by unknown factors. The debate was not ended by more detailed gene information in subsequent drafts of the chicken genome [8, 15] nor by the massive amount of information provided by more than 50 other avian genomes [3, 10]. True absence seems very unlikely for such a large number of genes, often encoding key mediators or regulators of function, with protein kinase A (catalytic subunit), leptin, p53, GLUT4 as examples. In fact, the intensive and large scale efforts to find avian leptin—which encountered numerous difficulties because of a very high gene GC content—resulted in characterization of a full-length avian coding sequence [20, 21]. Since then repeated efforts have characterized some of the “missing” avian genes, so that a broad biological gap slowly filled with bits of information [12, 35]. But until today, the number of missing genes in avian genomes (1026 in the eight analyzed species) is still staggering and poorly understood. Even after a time-consuming manual addition of genes based on homology and synteny, we observed that still 833 genes could not be found in any of the eight bird genomes (Additional file 3: Fig. S2). As we selected these genomes from a larger set of sequenced species on basis of the best annotation (highest number of annotated genes) the extension of our analysis of “missing” genes from 8 to 75 genomes did not diminish the phenomenon and it can be calculated that from these 75 genomes only more than 10exp5 gene sequences await to be discovered.

In this study, we have used a visualization method displaying characteristics such as transcript GC%, amino acid usage and nPD% of orthologous genes in function of genomic position [26]. This method provides a powerful tool to disclose regional effects of "missing" avian genes. The resulting genome-wide landscapes show that more than 2000 different genes are underrepresented (presence index < 0.70) in the avian databases and that these genes are clustered in fourteen discrete regions based on the human genome order. For 1026 genes no gene information was found in any bird genome in our analysis; 613 of those were located in these fourteen areas of “missing” gene clusters. So, as more than 70% (1517/2130) of the genes located in these “missing” gene clusters can be found in at least one bird species, there is indication of their presence also in other birds. The exceptional bird sequences that could be found in these regions allowed us to study some basic characteristics. First, evidence for the existence of sequencing refractory regions was the enrichment of gene fragments in the regions with "missing" genes. A second indication was the strong positive correlation between regions of "missing" genes and a rise of average DNA GC%, especially in bird genomes. This correlation is plausible, given the fact that GC rich sequence is particularly hard to characterize. Indeed, next-generation sequencing technologies suffer from a GC bias and show a low coverage of GC rich sequences and repeats [13, 14, 29]. Sequencing using the SMRT technology, which is more robust to GC content [36], showed an improvement of the quality of two songbird genomes [37]. However, “missing” genes are also underrepresented in these genomes. Due to problems with GC-rich genes and proper genome annotation, a well-defined avian karyotype is not available. We have displayed the data in the genome order of the most studied bird genome: the chicken. However, also in this species 19% of the genes (2633) are “missing” and for another 214 genes a gene location is absent (“position unknown”). Despite the large amount of missing gene information (sequence and position), we observed the highest GC content in the microchromosomes (Fig. 2b), which is in agreement with the literature [38–40]. As can be seen from the 214 genes which are present in the chicken genome but the position is unknown (PU) in Figs. 2, 3c and 4b, these genes have a high GC content and nPD%, similar to the microchromosomes. For the 1821 genes for which gene information is available in at least one bird, but of which no positional information is available in chicken, the GC content is 7% higher and the nPD% is 1.58 times higher as compared to the genes of which a position is known in chicken. It is likely that a substantial part of the genes which are not annotated, high in GC and fast evolving are located on microchromosomes. Indeed, according to NCBI genome, no genes are currently mapped on the microchromosomes 29, 34, 35, 36, 37 and 38.

A likely mechanism for a regional rise in GC content is GC-biased gene conversion (gBGC—reviewed in Ref. [41]). Ambiguities between paternal and maternal alleles in the heteroduplex DNA formed during meiotic recombination are repaired with a small bias in favor of the G or C over A or T. This small discrepancy can lead to large differences in GC content after thousands of generations. This process was first studied in yeast [42] and later in primates [43, 44], other mammals [45, 46], non-avian reptiles [47] and birds [48–50]. The mechanism of gBGC is particularly interesting given the well conserved karyotype of avian genomes: consisting of ~ 10 macrochromosomes and ~ 30 microchromosomes [51]. During meiosis, one recombination event must happen per chromosome before segregation. Thus: the smaller the chromosome, the higher the chance that gBGC has caused a mutation per unit of chromosome length [40, 49]. Moreover, avian microchromosomes have an increased genes density as together they contribute only 25% to genomic DNA content, but they encode about 50% of all avian genes [38–40]. The gene density on microchromosomes might well be even higher, as a result of the “hidden” genes which are preferentially associated with microchromosomes. Together, high gene density and small chromosome size increases the chance per generation that gBGC alters the GC content of the coding region of genes. Another influence of this mechanism on genome evolution, is that meiotic cross-over recombines mutations of neighboring genes into an epistatic complex that endows offspring with novel heritable characteristics.

The observed accelerated evolution and high GC content are two obstacles to allow proper sequencing and annotation of genes. It is of interest to see that the heatmap plots of normalized divergence rates of orthologous proteins, when ordered in the gene order of the chicken genome (Fig. 4b), show an overall gradient of low protein divergence in the macrochromosomes (except accelerations in some subtelomeric regions, e.g. the p-arm of chromosomes 1, 2, 3, 4, 5 and 10 and q-arm of chromosomes 1, 3, 4 and 10) to high protein divergence in the microchromosomes. Of interest is a high rate of protein divergence in the group of genes that are currently waiting to be mapped in the chicken genome (PU in Fig. 4b). Given the high GC%, it is likely that many of these genes will be mapped to microchromosomes, or subtelomeres of incompletely sequenced macrochromosomes. In line with the idea that a meiotic mechanism of GC-biased gene conversion is responsible for this accelerated protein evolution it is of interest to compare the nearly identical landscapes of GC% (Fig. 3c) and protein divergence rates (Fig. 4b).

The systematic genome-wide search for characteristics of the large amount of "missing" genes in avian genomes thus indicates that technical barriers of GC accumulation and accelerated protein divergence hinder the current efforts of complete genome sequencing and annotation. This fits with the notion that many of the "missing" bird genes must be present as they are needed for crucial vertebrate body functions [12, 17, 52]. One of those is energy metabolism in fast glycolytic type II muscle fibers, which are vital for life-and-death situations such as hunting or being hunted. In such fibers, a massive flux of glycolytic metabolites produces ATP for contraction. Both rapid glycogenolysis and rapid glucose uptake are required to boost glycolytic flux and the key flux controlling proteins for these metabolic steps are the muscle type glycogen phosphorylase PYGM, the insulin-regulated glucose transporter GLUT4 (gene *SLC2A4*). Moreover, both aldolase ALDOA and enolase ENO3 [53, 54] are key glycolytic enzymes with typical high expression in skeletal muscle. Indeed in vertebrates, all four proteins are particularly abundant in skeletal muscle and essential for the rapid anaerobic ATP production in type II muscle fibers. Most bird genomes lack information for *ALDOA, ENO3, PYGM* and *SLC2A4* and it is intriguing that each of the four genes encoding these proteins reside in one of the clusters of "missing" genes with high GC content that underwent accelerated protein evolution. The absence of GLUT4 (*SLC2A4*) in birds, despite intense research efforts is well known in the literature [55–59]. The major interest in this protein is not surprising, as GLUT4 plays an important role in mammalian glucose homeostasis, regulating uptake of glucose in adipose tissue, skeletal muscle and heart in response to insulin [60]. Glucose homeostasis shows large differences between mammals and birds [25] and a lack of GLUT4 could have been an explanation for this difference. Several researchers tried to identify other glucose transporters which compensate for the loss of GLUT4 in birds [56, 57, 61]. However, we believe that the absence of a GLUT4 gene is not the explanation for the high blood glucose and insulin resistance in birds. Indeed, we show evidence for expression of GLUT4 mRNA in the expected tissues; moreover, this mRNA is translated by skeletal muscle. However, GLUT4 immunoreactive protein could not be detected in chicken heart, despite an mRNA signal. This is in agreement with the observation that glucose is taken up in skeletal muscle but not in heart of insulin injected chicks [62]. We think that identification of GLUT4, both on mRNA as on the protein level, is a major and long awaited for milestone in the better understanding of glucose homeostasis in birds which is still enigmatic. For instance, many questions remain unanswered in the rapid metabolic transition from a fasted state combined with high levels of energy expenditure during long distance migratory flight to a phase of anabolism during a refueling stop [63]. Are insulin and GLUT4 involved to restore glycogen and triglyceride reserves in flight muscle? It is also poorly understood why on the one hand in humans fasting blood glucose concentrations above 7 mM is defined as diabetes mellitus, with its chronic complications when left untreated, while on the other hand birds thrive with blood glucose values that far exceed this threshold [25, 64]. Moreover, many bird species have a long life span despite a high metabolic rate [65] and maintain a high degree of fitness until the end of life [66]. Birds are also known to be resistant to the hypoglycemic effects of insulin [56, 58]. From a biomedical perspective it seems of interest to better understand how birds cope with a chronic state of insulin resistance and elevated blood glucose levels without developing diabetes and its complications.

Proof of principle for the idea of “hidden” genes in GC-rich DNA regions that are hard to sequence was extended by determining the full length mRNA transcripts of three other important genes related to the glucose metabolism in skeletal muscle. For *ALDOA*, *ENO3* and *PYGM* there were already some indications that the gene is present (as fragments) in the chicken genome [67–69]. Of interest is the observation that only the genes encoding muscle-type isoforms (*ALDOA*, *ENO3* and *PYGM*) and none of the other paralogs (*ALDOB*, *ALDOC*, *ENO1*, *ENO2*, *ENO4*, *PYGL*, *PYGB*) reside in one of the clusters of "missing" genes with high GC content. The same applies to the muscle-type glucose transporter *SLC2A4* (GLUT4) while the other paralogous genes (*SLC2A1, SLC2A2* and *SLC2A3*) are excluded from these GC-rich DNA regions. This raises the interesting possibility of an orchestrated accelerated protein evolution of a set of proteins that may have helped to evolve the special needs of energy production during flight and rapid transition during refueling.

## Conclusion

Our study of genome-wide landscapes of GC accumulation, amino acid composition and protein divergence has exposed a link between numerous “missing” genes clustered in GC-rich regions and accelerated evolution of the proteins encoded by these genes. Detection of mRNA and protein sequence delivers the proof of principle that physiologically important “missing” vertebrate genes are “hidden” by a GC-rich context in bird genomes and that these genes are expressed in chicken skeletal muscle. Moreover, by comparison of genomes from pairs of panther, bat and crocodile species we illustrate that "hidden" genes also cluster in GC-rich areas in non-avian vertebrate genomes. The influence of genomic position on the rate of protein evolution provides new perspectives to study macro-evolutionary events.

## Methods

### Retrieving of genome data

Data for GC content (GC%), amino acid usage (AA usage%) and protein divergence were retrieved for 14 vertebrate genomes including two reference genomes (*Homo sapiens* and *Lepisosteus oculatus*), *Alligator mississippiensis* (Archosauriformes), *Chrysmemys picta* (Testudines), *Pogona vitticeps* and *Python bivittatus* (Squamata) and eight bird genomes which were selected on basis of the highest number of annotated protein encoding genes and representatives of major taxa: two Paleognathae (*Apteryx australis* and *Struthio camelus*) and six Neognathae, with representatives from the clades of Galloanserae (*Anser cygnoides* and *Gallus gallus*), Passeriformes (*Pseudopodoces humilis* and *Sturnus vulgaris*), Accipitriformes (*Aquila chrysaetos*) and Caprimulgimorphae (*Calypte anna*). An extended set of 75 bird genomes (listed in Additional file 10: Table S5) was used for verification. A list of 15,135 common vertebrate protein encoding genes was defined on basis of presence in both the human genome as well as in at least one of the crocodile, lizard, turtle or snake genomes. In a manual analysis, we checked for uncounted genes that were indeed present under a different name (LOCnumber) using gene description, sequence homology and synteny as criteria. This enlarged the list of common vertebrate protein encoding genes from 15,135 to 15,624. To assess the generic relationship between regions with high GC% and increased occurrence of “missing” genes, we also studied the genomes of *Panthera pardus*, *Panthera tigris*, *Myotis brandtii*, *Myotis lucifugus* and *Crocodylus porosus*. Gene lists from which the figures were calculated can be accessed on the Open Science Framework via following link:

https://osf.io/sv7hb/?view_only=35a1a2d7ff7b45168b3cba1854314d47.

### Calculation of presence and length indices and presence score

We calculated the relative presence or presence index as follows: for each gene we counted how many times the gene was present in the bird genomes and this was divided by 8 (the total number of birds). Next a sliding window value was calculated for every gene and its 100 neighbors (50 at each side), as previously described [26]. For each gene of the list of common vertebrate protein encoding genes the length index of a transcript was calculated by dividing the average transcript length of the non-avian vertebrates (human, spotted gar, alligator, turtle, lizard and snake), by the average predicted transcript length encoded by the avian orthologs. A sliding window with 101 genes was used as for the presence index. Note that the transcript length of the birds is in the denominator: the shorter the average transcript length in birds, the higher the length index.

The presence score (used in Fig. 6 and Additional file 8: Fig. S4) was calculated by comparing the genes which are present in the genomes of two related species. If the gene is only present in the best annotated genome (*Panthera pardus* and *Alligator mississippiensis*), the gene got a score of − 1. When the gene is present in both species, the score is 0 and if the gene is only present in the less annotated genome (*Panthera tigris* and *Crocodylus porosus*) the score is + 1. A sliding window of 101 genes was applied to this metric and shown in the Figures.

### Normalized protein divergence

For each pair of orthologues protein sequences (corresponding to the XM numbers in Additional file 1: Table S1), the protein divergence was calculated using the EMBOSS Stretcher tool as previous described [26]. Data for which the protein identity was < 30% were not included. For each protein pair, the protein divergence was divided (normalized) by the average of all protein divergence rates for the two species in the comparison. This normalized protein divergence was abbreviated as nPD%.

### Animals, tissue dissection, RNA and cDNA

Experiments were approved by the ethical committee of the KU Leuven (project number 124/2012). Tissues were procured from decapitated female broilers (ISA Brown, age 27 weeks bred by Munckenei, Wingene, Belgium), rinsed in phosphate buffered saline and snap-frozen in liquid nitrogen. Samples were stored at − 80 °C until use. RNA was extracted from homogenized tissues using TRIzol (Thermo Fisher Scientific, Belgium), and reverse transcribed to cDNA (RevertAid H Minus cDNA synthesis kit, Thermo Fisher Scientific) with the recommendations for GC-rich sequences according to the manufacturer’s protocol. cDNA synthesis was performed using random hexamers or a gene specific primer or with a polyT-primer.

### Primers, PCR, cloning, sequencing and quantitative RT-PCR

Primers and probes were ordered from Sigma Aldrich (Belgium). A list of the used primers can be found in Additional file 6 and for the quantitative PCR in Additional file 9: Table S4. Several databases (NCBI: gene, nucleotide, SRA and uniprot) were screened for the presence of a (partial) sequence from chicken or an evolutionary related species (reptiles and other bird sequences). Sequences from databases were BLASTed against EST databases to find additional sequences. Alignments (using EMBOSS ClustalΩ) were performed and primers were developed based on the sequences that were generated and had maximal conservation among species. Also, data from RNA seq included in SRA files (SRR924561 and SRR924559) were used to extend the sequence and served as a template. More details are described in Additional file 6.

Classic PCR and/or nested PCR or 3′ Rapid Amplification of cDNA Ends (3’RACE) was performed using the AccuPrime GC Rich polymerase (Thermo Fisher Scientific). We used buffer B (for the GC rich sequences) and 100 ng cDNA as template, according to manufacturer’s protocol. PCR products were separated on a 1.5% agarose gel and purified using GeneClean Turbo kit (MP Biomedicals, Belgium). After purification, the PCR product was cloned into pGEM-T-Easy vector (Promega, The Netherlands) and transformed into DH5α *E. coli* competent cells. The purified plasmid was isolated from *DH5α E. coli* using GeneJET Plasmid Miniprep kit (Thermo Fisher Scientific) and sent for Sanger sequencing (LGC Genomics, Berlin). Quantitative RT-PCR was performed on 5 ng cDNA [70] to study the expression of *ALDOA, PYGM, ENO3* and *SLC2A4* by using ABsolute qPCR mix (Westburg, The Netherlands) on a Rotor-Gene 3000 (Corbett Research) on different chicken tissues (n = 3). Calculations of the expression were based on the Pfaffl method [71] after normalization with *RPS13*. The expression signals shown are relative to expression in the pectoralis muscle. A list of the used primers and probes can be found in Additional file 9: Table S4.

### Measurement of immunoreactive GLUT4

A peptide (LRGPTPRMGVLRLLGSPRL) corresponding to amino acids 262–280 in the large cytoplasmic loop of chicken GLUT4 was ordered from EZBiolab (Indiana, United States). This peptide was coupled to keyhole limpet haemocyanin using the Imject™ Maleimide-Activated mcKLH Spin Kit (Thermo Fisher Scientific) and injected to New Zealand White rabbits [72]. Crude antiserum was affinity purified using the peptide coupled to bovine serum albumin using the Imject™ Maleimide-Activated BSA Spin Kit (Thermo Fisher Scientific). GAPDH antibody was purchased from Abcam (ab8245, UK). Secondary antibodies coupled to peroxidase were used for detection: anti-rabbit IgG antibody (NA934V, GE Healthcare, Belgium) and anti-mouse IgG antibody (715-036-150, Jackson Laboratory, West Grove, USA).

Total protein lysates were made of the different tissues as previous described [70]. Lysates of the cytosolic protein fraction were obtained with the Plasma membrane extraction kit (Abcam, UK) in step A6 (different fractions including total cellular membrane and plasma membrane were obtained). Total protein lysates (30 µg) were separated on 4–12% Criterion XT Bis–Tris Protein Gel (Bio-Rad) with MOPS (3-(*N*-morpholino) propanesulfonic acid) solution. Hereafter, proteins were transferred to polyvinylidene fluoride membrane (GE Healthcare) and non-specific binding was blocked overnight with 5% milk in Tris buffered saline with 0.05% Tween20. Primary antibody was diluted 1/1,000 (anti-GLUT4) or 1/5,000 (anti-GAPDH) in 1% milk and the membranes were incubated for 1 h at room temperature. Secondary antibodies was diluted 1/2000 (anti-rabbit IgG) or 1/5000 (anti-mouse IgG) and incubated with the membrane for 1 h at room temperature. Detection was done using Pierce ECL Western Blotting Substrate (Thermo Fisher Scientific) and an Agfa Curix developing machine.

### Mass spectrometry

For ALDOA, ENO3 and PYGM: a piece of 300 mg chicken pectoralis muscle was homogenized in 600 µL buffer the Plasma Membrane Extraction kit (Abcam) to obtain the cytosolic fraction. This fraction was subjected to protein precipitation [73], dissolved in 8 M urea in 50 mM Tris (pH 7.7), and digested for 4 h at 37 °C with 0.5 µg endolysC-trypsin (Promega). The sample was subsequently diluted four times and left overnight at 37 °C. For GLUT4, Magnetic Dynabeads (Thermo Fisher Scientific) were coupled to 35 µg of the anti-chicken GLUT4 antibody according to the manufacturer’s protocol. These beads were added to total protein lysates (200 mg in 2 mL S1 buffer [70]) and incubated for 4 h at 4 °C while rotating). After incubation, the beads were washed 3 times with MilliQ water. Next, the beads were eluted with 50 µL of 5% acetic acid by incubation for 5 min at room temperature. The eluate was transferred to a new tube containing 50 µl 1 M Tris. After twofold dilution, the sample was subjected to an overnight trypsin (Promega) (1 µg) digestion at 37 °C. The resulting peptides were desalted using C18 ZipTip pipette tips (Millipore, France) and subjected to high-resolution LC–MS/MS using an Ultimate 3000 nano UPLC system interfaced with a Q Exactive hybrid quadrupole-orbitrap mass spectrometer via an EASY-spray (C-18, 15 cm) column (Thermo Fisher Scientific). Peptides were identified by MASCOT (Matrix Science) using a custom database (all chicken Refseq proteins plus the predicted protein sequences from ALDOA, ENO3, PYGM and GLUT4; total of 39,235 proteins) via Proteome Discoverer 2.2 software, incorporating Percolator for peptide validation. Oxidation (M) was used as a variable modification. Two missed cleavages were allowed for trypsin digestion. Peptide tolerance was set at 10 ppm and MS/MS tolerance at 20 mmu. Only peptides with a high confidence (posterior error probability < 0.01) were taken into account.

### Statistics

R was used for analysis, and except where mentioned otherwise, base R statistical functions were used. Correlations were calculated using the Pearson product-moment correlation coefficient. To calculate significance of observed presence and length indices, 1000 randomized datasets were generated by scrambling the gene order. Shapiro–Wilk normality tests were used to validate normality of distributions. Normality for datasets were assumed with p-values < 0.01. To compare distributions, an empirical cumulative distribution function was calculated on the randomized datasets to calculate the threshold at which the observed data would have a probability ≤ 0.001 to derive from a random distribution. The same empirical cumulative distribution function, with assumption of random sampling with replacement, was used to calculate the probability of selecting a dataset with X values under or above the threshold index.

## Supplementary Information

**Additional file 1: Table S1.** List of used species and transcript ID. Manual additions were noted with an extra 1 in the adjacent column. Manual additions were done by looking at gene description and synteny.

**Additional file 2: Figure S1.** Distribution of presence and length index in sliding window and random generated. **a** Comparison of the distribution of the 15,135 sliding window values of the presence index (Fig. 1a—red) to the values of 1,000 random sets of 101 non-ordered genes (grey). Only one of the random sets (0.1%) had a presence index below 0.70, while with the genes ordered according to the human genome 2130 windows had a presence index below this threshold. **b** Comparison of the distribution of the 15,135 sliding window values of the length index (Fig. 1a—blue) to the values of 1000 random sets of 101 non-ordered genes (grey). The highest random set value was 1.46; 1214 genes of the sliding window approach exceed the threshold. **c** Venn diagram showing the large overlap between the windows that exceeded the 0.1% frequency threshold for presence and length.

**Additional file 3: Figure S2.** Effect of manual additions and extension of the number of studied bird genomes to the landscapes of the presence and length index. **a**. In each genome we verified manually whether "missing" genes were in fact present in the genome databases but under a different name than the standard gene name (or a LOCnumber). This resulted in an extended common vertebrate gene set with 15,624 genes (i.e. 489 more than the fully automated set of 15,135 genes). **b.** The landscapes are very similar using the manually curated and automated methods. This means that, despite this manual curation, the phenomenon of 14 regions in the human genome, in which bird have less genes annotated, the genes are still present as partial sequences. **c**. The clustering of “missing genes” and of “missing gene fragments” was reanalyzed in a set of 75 avian genome instead of 8 with the outcome that the clustered absence of gene information (minima in the missing gene index and maxima of the length index) does not change.

**Additional file 4: Figure S3.** Avian and reptilian landscapes presented on the genome of *Lepisosteus oculatus*. The same data as shown in Fig. 1a–d are here shown in the order of the *Lepisosteus oculatus* genome. **a** presence (red) and length (blue) indices. **b** GC content landscapes of *Pseudopodoces humilis* and *Chrysemys picta*. **c** and **d** landscapes of amino acid usage of the predicted protein sequence in *Pseudopodoces humilis* (**c**) and *Chrysemys picta* (**d**).

**Additional file 5: Table S2.** List of the used species pairs presented in the heatmap. The number corresponds to the number in the heatmaps.

**Additional file 6: **Extra information regarding the cloning of *ALDOA*, *ENO3*, *PYGM* and *SLC2A4*.

**Additional file 7: Table S3.** List of identified peptides using LC–MS/MS

**Additional file 8: Figure S4.** “Missing” genes in the *Crocodylus porosus* genome. The same data are shown as in Fig. 6c, but the genes are ranked in the order of the chicken genome. Note the relationship between “missing” genes and high GC content.

**Additional file 9: Table S4.** List of used primers and probes for quantitative PCR

**Additional file 10: Table S5.** List of the 75 studied avian genomes

**Additional file 11: Figure S5.** Complete images of the immunoblots of GLUT4 and glyceraldehyde-3-phosphate dehydrogenase (GAPDH), from which the essential information is shown in Fig. 5b.

## Data Availability

Data were extracted from publicly available genbank files sourced from NCBI. Further datasets generated and/or analyzed during the current study are available from the corresponding author on reasonable request. A list of the used XM-numbers (including the manual additions) is provided in Additional file 1: Table S1.

## Abbreviations

GC%: GC content
GARP%: Amount of amino acids glycine (G), alanine (A), arginine (R) and proline (P) in the protein
FYMINK%: Amount of amino acids phenylalanine (F), tyrosine (Y), methionine (M), isoleucine (I), glutamine (N) and lysine (K) in the protein
gBGC: GC-biased gene conversion
nPD%: Normalized protein divergence %
HS: *Homo sapiens*
LO: *Lepisosteus oculatus*
PU: Position unknown
Chr.: Chromosome

## References

[1] Zhou Z, Barrett PM, Hilton J (2003). An exceptionally preserved Lower Cretaceous ecosystem. Nature.

[2] Brusatte SL, O’Connor JK, Jarvis ED (2015). The origin and diversification of birds. Curr Biol.

[3] Zhang G, Li C, Li Q, Li B, Larkin DM, Lee C (2014). Comparative genomics reveals insights into avian genome evolution and adaptation. Science.

[4] Jarvis ED, Mirarab S, Aberer AJ, Li B, Houde P, Li C (2014). Whole-genome analyses resolve early branches in the tree of life of modern birds. Science.

[5] Carpenter KJ, Sutherland B (1995). Eijkman’s contribution to the discovery of vitamins. J Nutr.

[6] Hillier LW, Miller W, Birney E, Warren W, Hardison RC, Ponting CP (2004). Sequencing and comparative analysis of the chicken genome provide unique perspectives on vertebrate evolution. Nature.

[7] Scanes CG (2007). The global importance of poultry. Poult Sci.

[8] Warren WC, Hillier LW, Tomlinson C, Minx P, Kremitzki M, Graves T, et al. A new chicken genome assembly provides insight into avian genome structure. G3 (Bethesda). 2017;7:109–17. 10.1534/g3.116.035923 PMC5217101 27852011

[9] Feng S, Stiller J, Deng Y, Armstrong J, Fang Q, Reeve AH (2020). Dense sampling of bird diversity increases power of comparative genomics. Nature.

[10] Lovell PV, Wirthlin M, Wilhelm L, Minx P, Lazar NH, Carbone L (2014). Conserved syntenic clusters of protein coding genes are missing in birds. Genome Biol.

[11] Howe K, Clark MD, Torroja CF, Torrance J, Berthelot C, Muffato M (2013). The zebrafish reference genome sequence and its relationship to the human genome. Nature.

[12] Hron T, Pajer P, Pačes J, Bartůněk P, Elleder D (2015). Hidden genes in birds. Genome Biol.

[13] Ross MG, Russ C, Costello M, Hollinger A, Lennon NJ, Hegarty R (2013). Characterizing and measuring bias in sequence data. Genome Biol.

[14] Wang W, Wei Z, Lam T-W, Wang J (2011). Next generation sequencing has lower sequence coverage and poorer SNP-detection capability in the regulatory regions. Sci Rep.

[15] Thomas S, Underwood JG, Tseng E, Holloway AK (2014). Long-read sequencing of chicken transcripts and identification of new transcript isoforms. PLoS ONE.

[16] Botero-Castro F, Figuet E, Tilak M, Nabholz B, Galtier N. Avian genomes revisited: hidden genes uncovered and the rates vs. traits paradox in birds. Mol Biol Evol. 2017;34(March):3123–31. 10.1093/molbev/msx236 28962031

[17] Yin ZT, Zhu F, Lin F Bin, Jia T, Wang Z, Sun DT, et al. Revisiting avian “missing” genes from de novo assembled transcripts. BMC Genomics. 2019;20:1–10. 10.1186/s12864-018-5407-1 PMC6321700 30611188

[18] Zhang Y, Proenca R, Maffei M, Barone M, Leopold L, Friedman JM (1994). Positional cloning of the mouse obese gene and its human homologue. Nature.

[19] Halaas JL, Gajiwala KS, Maffei M, Cohen SL, Chait BT, Rabinowitz D (1995). Weight-reducing effects of the plasma protein encoded by the obese gene. Science.

[20] Seroussi E, Cinnamon Y, Yosefi S, Genin O, Smith JG, Rafati N (2016). Identification of the long-sought leptin in chicken and duck: expression pattern of the highly GC-Rich avian leptin fits an autocrine/paracrine rather than endocrine function. Endocrinology.

[21] Seroussi E, Pitel F, Leroux S, Morisson M, Bornelöv S, Miyara S (2017). Mapping of leptin and its syntenic genes to chicken chromosome 1p. BMC Genet.

[22] Friedman-Einat M, Seroussi E (2019). Avian leptin: bird’s-eye view of the evolution of vertebrate energy-balance control. Trends Endocrinol Metab.

[23] Mueckler M, Thorens B (2013). The SLC2 (GLUT) family of membrane transporters. Mol Aspects Med.

[24] Dupont J, Derouet M, Simon J, Taouis M (1998). Nutritional state regulates insulin receptor and IRS-1 phosphorylation and expression in chicken. Am J Physiol.

[25] Braun EJ, Sweazea KL (2008). Glucose regulation in birds. Comp Biochem Physiol B.

[26] Huttener R, Thorrez L, In’t Veld T, Granvik M, Snoeck L, Van Lommel L, et al. GC content of vertebrate exome landscapes reveal areas of accelerated protein evolution. BMC Evol Biol. 2019;19:144. 10.1186/s12862-019-1469-1 PMC6636035 31311498

[27] Lovell PV, Wirthlin M, Carbone L, Warren WC, Mello CV (2015). Response to Hron et al.. Genome Biol.

[28] Bornelöv S, Seroussi E, Yosefi S, Pendavis K, Burgess SC, Grabherr M (2017). Correspondence on Lovell et al.: identification of chicken genes previously assumed to be evolutionarily lost. Genome Biol.

[29] Benjamini Y, Speed TP (2012). Summarizing and correcting the GC content bias in high-throughput sequencing. Nucleic Acids Res.

[30] Carlberg C, Quaas R, Hahn U, Wittig B (1987). Sequencing refractory GC rich regions in plasmid DNA. Nucleic Acids Res.

[31] Shewan AM, Marsh BJ, Melvin DR, Martin S, Gould GW, James DE (2000). The cytosolic C-terminus of the glucose transporter GLUT4 contains an acidic cluster endosomal targeting motif distal to the dileucine signal. Biochem J.

[32] Martinez-Arca S, Lalioti VS, Sandoval IV (2000). Intracellular targeting and retention of the glucose transporter GLUT4 by the perinuclear storage compartment involves distinct carboxyl-tail motifs. J Cell Sci.

[33] Melvin DR, Marsh BJ, Walmsley AR, James DE, Gould GW. Analysis of amino and carboxy terminal glut-4 targeting motifs in 3T3-L1 adipocytes using an endosomal ablation technique. Biochemistry. 1999;38:1456–62. 10.1021/bi980988y 9931010

[34] Blot V, Mcgraw TE. Molecular mechanisms controlling GLUT4 intracellular retention. Mol Biol Cell. 2008;19(August):3477–87. 10.1091/mbc.E08-03-0236 PMC2488284 18550797

[35] Denyer MP, Pinheiro DY, Garden OA, Shepherd AJ. Missed, not missing: phylogenomic evidence for the existence of avian FoxP3. PLoS ONE. 2016;11:e0150988. 10.1371/journal.pone.0150988 PMC4777427 26938477

[36] Shin SC, Ahn DH, Kim SJ, Lee H, Oh T-J, Lee JE, et al. Advantages of single-molecule real-time sequencing in high-GC content genomes. PLoS ONE. 2013;8:e68824. 10.1371/journal.pone.0068824 PMC3720884 23894349

[37] Korlach J, Gedman G, Kingan SB, Chin CS, Howard JT, Audet JN (2017). De novo PacBio long-read and phased avian genome assemblies correct and add to reference genes generated with intermediate and short reads. Gigascience.

[38] Smith J, Bruley CK, Paton IR, Dunn I, Jones CT, Windsor D (2000). Differences in gene density on chicken macrochromosomes and microchromosomes. Anim Genet.

[39] Burt DW (2002). Origin and evolution of avian microchromosomes. Cytogenet Genome Res.

[40] Axelsson E, Webster MT, Smith NGC, Burt DW, Ellegren H (2005). Comparison of the chicken and turkey genomes reveals a higher rate of nucleotide divergence on microchromosomes than macrochromosomes. Genome Res.

[41] Duret L, Galtier N (2009). Biased gene conversion and the evolution of mammalian genomic landscapes. Annu Rev Genomics Hum Genet.

[42] Birdsell JA (2002). Integrating genomics, bioinformatics, and classical genetics to study the effects of recombination on genome evolution. Mol Biol Evol.

[43] Dreszer TR, Wall GD, Haussler D, Pollard KS (2007). Biased clustered substitutions in the human genome: the footprints of male-driven biased gene conversion. Genome Res.

[44] Berglund J, Pollard KS, Webster MT. Hotspots of biased nucleotide substitutions in human genes. PLoS Biol. 2009;7:e26. 10.1371/journal.pbio.1000026 PMC2631073 19175294

[45] Romiguier J, Ranwez V, Douzery EJP, Galtier N (2010). Contrasting GC-content dynamics across 33 mammalian genomes: relationship with life-history traits and chromosome sizes. Genome Res.

[46] Capra JA, Pollard KS (2011). Substitution patterns are GC-biased in divergent sequences across the metazoans. Genome Biol Evol.

[47] Figuet E, Ballenghien M, Romiguier J, Galtier N (2014). Biased gene conversion and GC-content evolution in the coding sequences of reptiles and vertebrates. Genome Biol Evol.

[48] Pessia E, Popa A, Mousset S, Rezvoy C, Duret L, Marais GAB (2012). Evidence for widespread GC-biased gene conversion in eukaryotes. Genome Biol Evol.

[49] Ellegren H (2013). The evolutionary genomics of birds. Annu Rev Ecol Evol Syst.

[50] Weber CC, Boussau B, Romiguier J, Jarvis ED, Ellegren H (2014). Evidence for GC-biased gene conversion as a driver of between-lineage differences in avian base composition. Genome Biol.

[51] O’Connor RE, Kiazim L, Skinner B, Fonseka G, Joseph S, Jennings R (2019). Patterns of microchromosome organization remain highly conserved throughout avian evolution. Chromosoma.

[52] Laine VN, Gossmann TI, van Oers K, Visser ME, Groenen MAM (2019). Exploring the unmapped DNA and RNA reads in a songbird genome. BMC Genomics.

[53] Baqué S, Guinovart JJ, Gómez-Foix AM (1996). Overexpression of muscle glycogen phosphorylase in cultured human muscle fibers causes increased glucose consumption and nonoxidative disposal. J Biol Chem.

[54] Richter EA, Hargreaves M. Exercise, GLUT4, and skeletal muscle glucose uptake. Physiol Rev. 2013;93:993–1017. 10.1152/physrev.00038.2012 23899560

[55] Carver FM, Shibley IA, Pennington JS, Pennington SN (2001). Differential expression of glucose transporters during chick embryogenesis. Cell Mol Life Sci C.

[56] Seki Y, Sato K, Kono T, Abe H, Akiba Y (2003). Broiler chickens (Ross strain) lack insulin-responsive glucose transporter GLUT4 and have GLUT8 cDNA. Gen Comp Endocrinol.

[57] Kono T, Nishida M, Nishiki Y, Seki Y, Sato K, Akiba Y (2005). Characterisation of glucose transporter (GLUT) gene expression in broiler chickens. Br Poult Sci.

[58] Sweazea KL, Braun EJ (2006). Glucose transporter expression in English sparrows (*Passer domesticus*). Comp Biochem Physiol B.

[59] Welch KC, Allalou A, Sehgal P, Cheng J, Ashok A. Glucose transporter expression in an avian nectarivore: the ruby-throated hummingbird (*Archilochus colubris*). PLoS ONE. 2013;8:e77003. 10.1371/journal.pone.0077003 PMC3796544 24155916

[60] Birnbaum MJ, GLUT-4. Identification of a novel gene encoding an insulin-responsive glucose transporter protein. Cell. 1989;57:305–15. 10.1016/0092-8674(89)90968-9 2649253

[61] Coudert E, Pascal G, Dupont J, Simon J, Cailleau-Audouin E, Crochet S (2015). Phylogenesis and biological characterization of a new glucose transporter in the chicken (*Gallus gallus*), GLUT12. PLoS ONE.

[62] Tokushima Y, Takahashi K, Sato K, Akiba Y (2005). Glucose uptake in vivo in skeletal muscles of insulin-injected chicks. Comp Biochem Physiol B.

[63] Jenni-Eiermann S (2017). Energy metabolism during endurance flight and the post-flight recovery phase. J Comp Physiol A.

[64] Simon J, Guillaumin S, Chevalier B, Derouet M, Guy G, Marche G (2000). Plasma glucose-insulin relationship in chicken lines selected for high or low fasting glycaemia. Br Poult Sci.

[65] Munshi-South J, Wilkinson GS (2010). Bats and birds: exceptional longevity despite high metabolic rates. Ageing Res Rev.

[66] Ricklefs RE (2010). Insights from comparative analyses of aging in birds and mammals. Aging Cell.

[67] Schettino CM, Lima DF, Leyton JF, El-Dorry HA, Bacila M (1981). Studies on the structure of aldolase A from chicken muscle. Biochim Biophys Acta.

[68] Tanaka M, Maeda K, Nakashima K (1995). Chicken α-enolase but not β-enolase has a src-dependent tyrosine-phosphorylation site: cDNA cloning and nucleotide sequence analysis. J Biochem.

[69] Sokolove PM (1985). Altered membrane association of glycogen phosphorylase in the dystrophic chicken. Biochim Biophys Acta.

[70] Lemaire K, Moura RF, Granvik M, Igoillo-Esteve M, Hohmeier HE, Hendrickx N, et al. Ubiquitin fold modifier 1 (UFM1) and its target UFBP1 protect pancreatic beta cells from ER stress-induced apoptosis. PLoS ONE. 2011;6:e18517. 10.1371/journal.pone.0018517 PMC3071830 21494687

[71] Pfaffl MW (2001). A new mathematical model for relative quantification in real-time RT-PCR. Nucleic Acids Res.

[72] Hu YX, Guo JY, Shen L, Chen Y, Zhang ZC, Zhang YL (2002). Get effective polyclonal antisera in one month. Cell Res.

[73] Wessel D, Flügge UI (1984). A method for the quantitative recovery of protein in dilute solution in the presence of detergents and lipids. Anal Biochem.

[74] Thorrez L, Van Deun K, Tranchevent L-C, Van Lommel L, Engelen K, Marchal K, et al. Using ribosomal protein genes as reference: a tale of caution. PLoS ONE. 2008;3:e1854. 10.1371/journal.pone.0001854 PMC2267211 18365009

