# Supplementary figures and images for: Sequencing refractory regions in bird genomes are hotspots for accelerated protein evolution
Source: BMC Ecol Evol. 2021 Sep 18;21:176. doi: 10.1186/s12862-021-01905-7 (PMC8449477; doi:10.1186/s12862-021-01905-7)

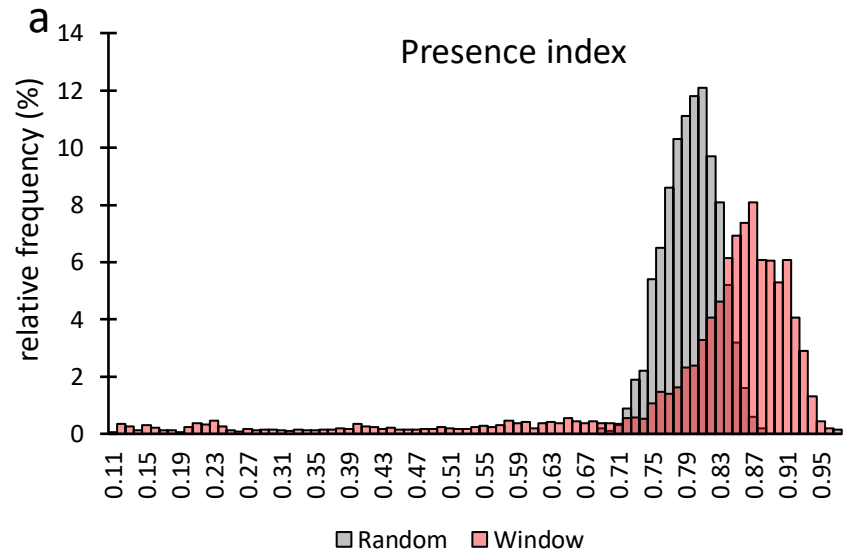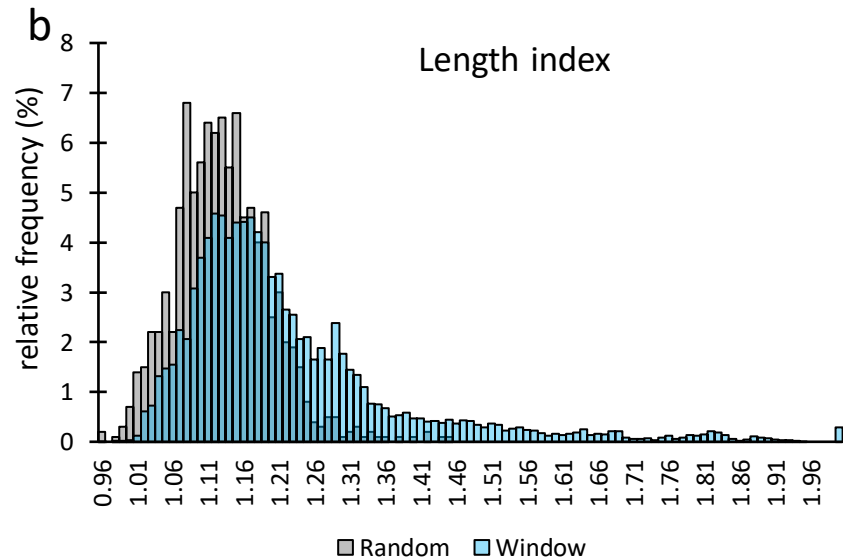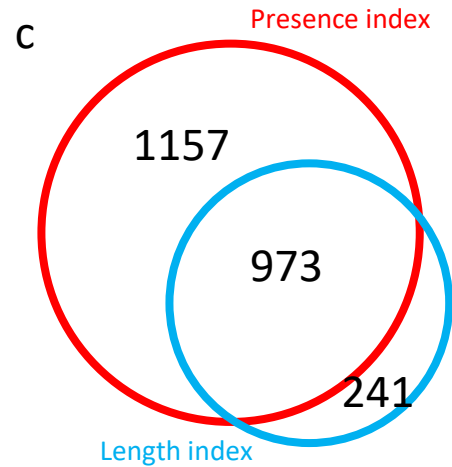

Supplement: Supplementary file 2 — Additional file 2: Figure S1. Distribution of presence and length index in sliding window and random generated. a Comparison of the distribution of the 15,135 sliding window values of the presence index (Fig. 1a—red) to the values of 1,000 random sets of 101 non-ordered genes (grey). Only one of the random sets (0.1%) had a presence index below 0.70, while with the genes ordered according to the human genome 2130 windows had a presence index below this threshold. b Comparison of the distribution of the 15,135 sliding window values of the length index (Fig. 1a—blue) to the values of 1000 random sets of 101 non-ordered genes (grey). The highest random set value was 1.46; 1214 genes of the sliding window approach exceed the threshold. c Venn diagram showing the large overlap between the windows that exceeded the 0.1% frequency threshold for presence and length. [file 12862_2021_1905_MOESM2_ESM.pdf]

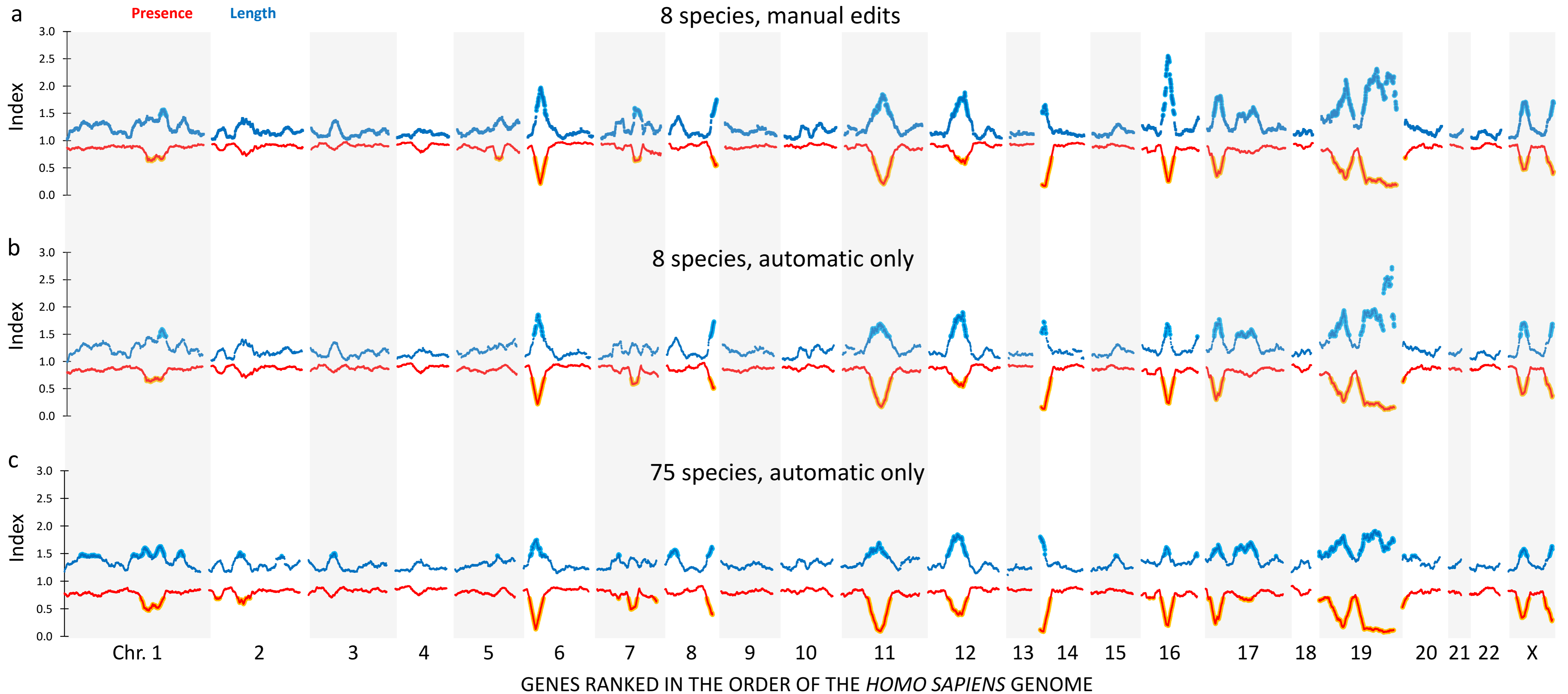

Supplement: Supplementary file 3 — Additional file 3: Figure S2. Effect of manual additions and extension of the number of studied bird genomes to the landscapes of the presence and length index. a. In each genome we verified manually whether "missing" genes were in fact present in the genome databases but under a different name than the standard gene name (or a LOCnumber). This resulted in an extended common vertebrate gene set with 15,624 genes (i.e. 489 more than the fully automated set of 15,135 genes). b. The landscapes are very similar using the manually curated and automated methods. This means that, despite this manual curation, the phenomenon of 14 regions in the human genome, in which bird have less genes annotated, the genes are still present as partial sequences. c. The clustering of “missing genes” and of “missing gene fragments” was reanalyzed in a set of 75 avian genome instead of 8 with the outcome that the clustered absence of gene information (minima in the missing gene index and maxima of the length index) does not change. [file 12862_2021_1905_MOESM3_ESM.pdf]

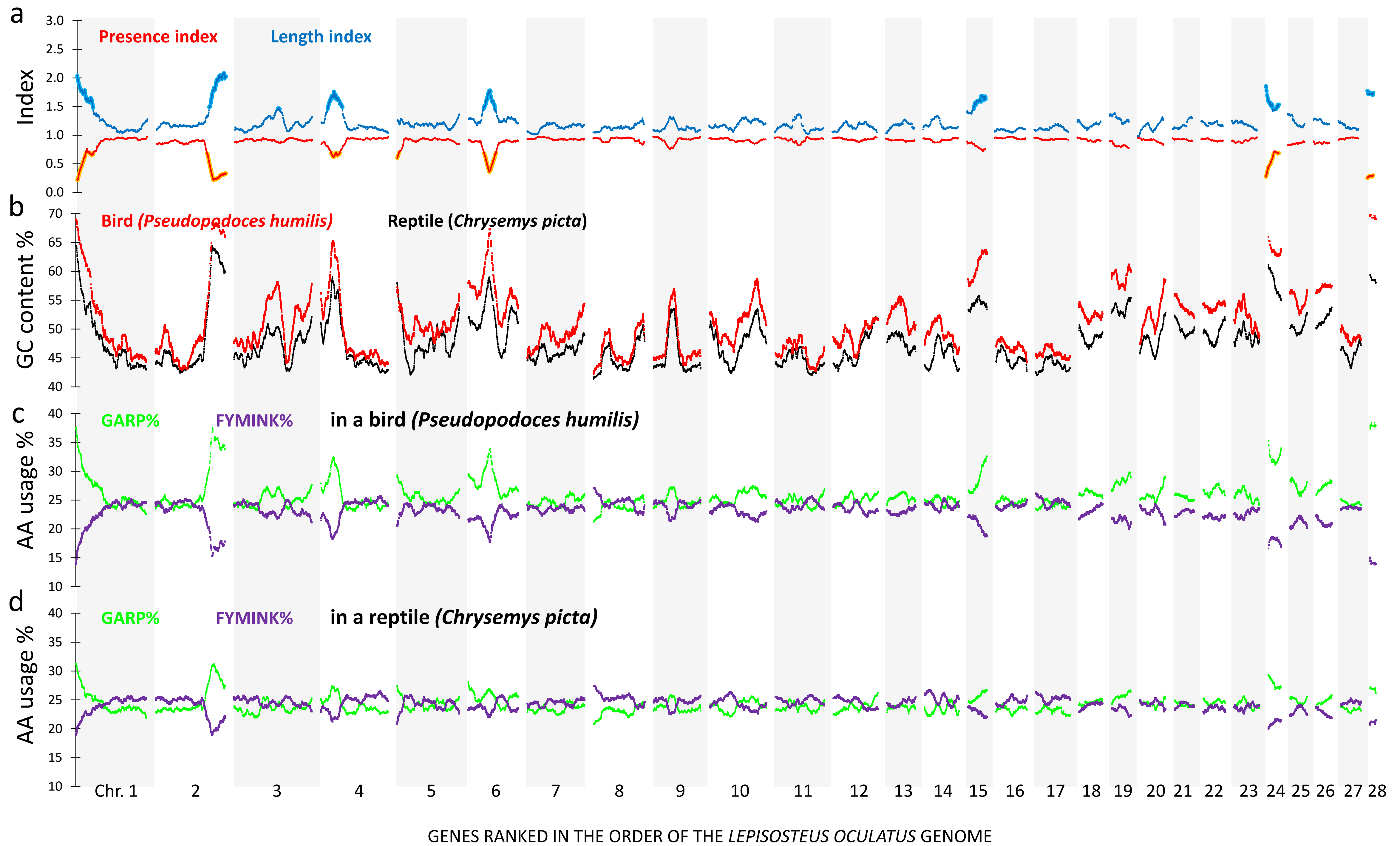

Supplement: Supplementary file 4 — Additional file 4: Figure S3. Avian and reptilian landscapes presented on the genome of Lepisosteus oculatus. The same data as shown in Fig. 1a–d are here shown in the order of the Lepisosteus oculatus genome. a presence (red) and length (blue) indices. b GC content landscapes of Pseudopodoces humilis and Chrysemys picta. c and d landscapes of amino acid usage of the predicted protein sequence in Pseudopodoces humilis (c) and Chrysemys picta (d). [file 12862_2021_1905_MOESM4_ESM.pdf]

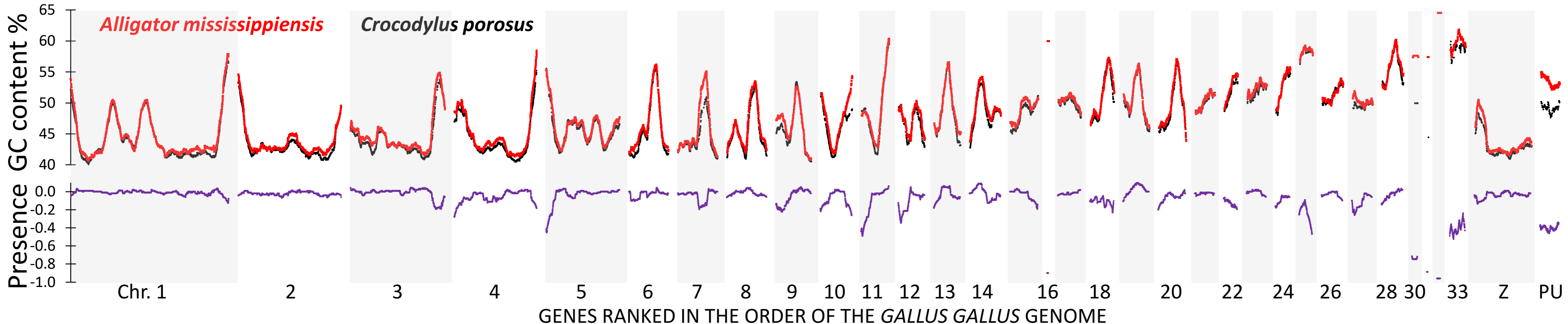

Supplement: Supplementary file 8 — Additional file 8: Figure S4. “Missing” genes in the Crocodylus porosus genome. The same data are shown as in Fig. 6c, but the genes are ranked in the order of the chicken genome. Note the relationship between “missing” genes and high GC content. [file 12862_2021_1905_MOESM8_ESM.pdf]

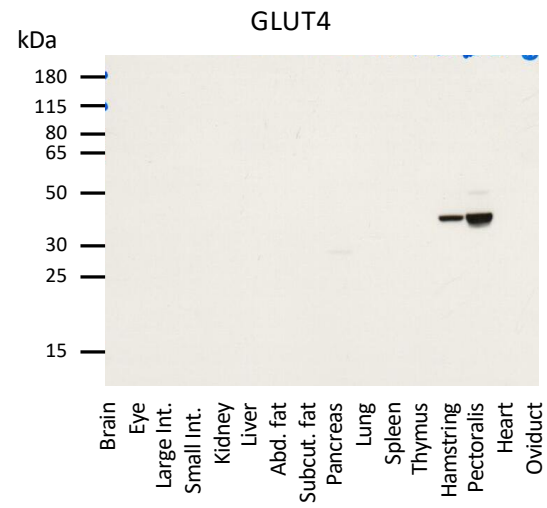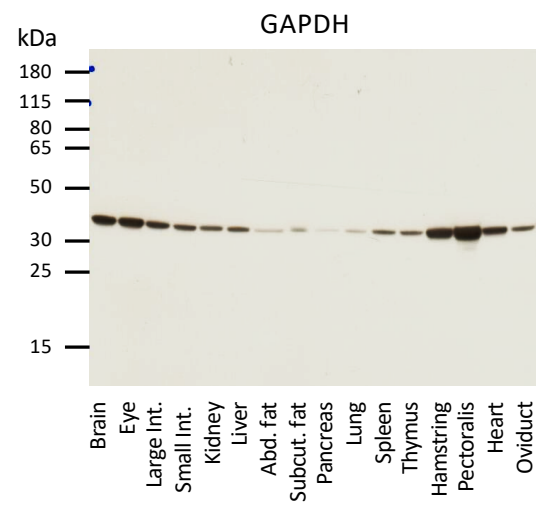

Supplement: Supplementary file 11 — Additional file 11: Figure S5. Complete images of the immunoblots of GLUT4 and glyceraldehyde-3-phosphate dehydrogenase (GAPDH), from which the essential information is shown in Fig. 5b. [file 12862_2021_1905_MOESM11_ESM.pdf]
