# Supplementary material for: Sequencing refractory regions in bird genomes are hotspots for accelerated protein evolution
Source: BMC Ecol Evol. 2021 Sep 18;21:176. doi: 10.1186/s12862-021-01905-7 (PMC8449477; doi:10.1186/s12862-021-01905-7)
Supplement: Supplementary file 6 — Additional file 6: Extra information regarding the cloning of ALDOA, ENO3, PYGM and SLC2A4. [file 12862_2021_1905_MOESM6_ESM.docx]

**Cloning of aldolase A (*ALDOA*)**

A first example of a gene that should be present but could not be found in the public databases (NCBI gene/nucleotide, Ensembl and Uniprot) is aldolase A (*ALDOA*). This protein was already described in 1981 and only a peptide of a muscle specific aldolase could be found [1]. Until now, the presence of the *ALDOA* gene in birds is underrepresented in the NCBI gene database. A sequence of *ALDOA* could be found in *Falco cherrug* (XM_005433403) and *Pseudopodoces humilis* (XM_005534075) and served as a template to clone chicken *ALDOA*. Based on these sequences, primers were developed and PCR using AccuPrime GC rich polymerase (Thermo Fisher – follow protocol for GC rich sequences) was executed. We found an mRNA sequence with a GC content of 65%. The sequence showed an identity of 81% with the human mRNA sequence and the predicted protein was 92% identical to the human protein sequence. At the start of the project, the sequence could only be found in the two mentioned birds above, but it can now also be found in *Anser cygnoides* (XM_013170655).

pBLAST of our own identified chicken *ALDOA* sequence to the human proteome revealed human *ALDOA* as the most related protein, confirming the correct isoform. In Fig. 5a the mRNA expression in different tissues is shown. Here we show a tissue-specific expression of *ALDOA* in muscle, which is in agreement with the mammalian expression profile.

The coding sequence of chicken *ALDOA* can be found at the end of the file.

**Enolase 3**

The gene encoding for muscle specific beta-enolase could not be found in the database (NCBI gene/nucleotide, Ensembl and Uniprot), despite the identification of the complete beta-enolase amino acid sequence in 1986 [2]. This is an example of a gene that should be there (because the protein is present in chicken), but a DNA/mRNA sequence was long absent from the databases. Remarkably, the mRNA sequence was already published in 1995 [3]. More than 20 years after the publication, the sequence could be found in the NCBI gene database (NM_205119), but is now not annotated in the current genome release. Based on this older sequence, primers were developed for quantitative PCR. The mRNA expression profile is given in Fig. 5a, showing a muscle specific expression profile.

**Cloning of glycogen phosphorylase (*PYGM*)**

Glycogen phosphorylase assays on chicken muscle are already performed for more than 30 years [4]. Despite this long time, the gene encoding for glycogen phosphorylase in the chicken cannot be found in the databases (NCBI gene/nucleotide, Ensembl and Uniprot). At the start, the only avian sequence could be found in *Pseudopodoces humilis*. Today, the sequence can also be found in *Apteryx australis* (XM_013943412), but it is “absent” in the other 6 out of 8 birds in our analysis. Here we show the cloning of chicken *PYGM*. Primers were developed, based on the sequence of *Pseudopodoces humilis* (XM_005533220), leading to the identification of a partial sequence of the chicken *PYGM* (using the AccuPrime polymerase). This newly identified sequence was BLASTed against the chicken EST database resulting in two hits (GGEZCB1010G06 and GGEZCB1001H04). Primers were developed based on the EST-sequences and PCR was performed. The GC content of the sequence was 62%. The mRNA sequence is 78% identical to the human sequence and the predicted protein is 86% identical to the human protein, confirming the identification.

An update on the NCBI-server (14-01-2019) showed a partial fragment of the *PYGM*-gene (LOC107049660) in chicken, corresponding to 164/843 amino acids in our identified sequence. Analogues to *ALDOA*, the chicken *PYGM* gene was most related to the human isoform and the expression profile is given in Fig. 5a, confirming the muscle specific expression of the glycogen phosphorylase gene in chickens. The mRNA sequence can be found at the end of the file.

**Cloning of glucose transporter 4 (GLUT4 or SLC2A4)**

A fourth example to strengthen our data was the cloning of avian glucose transporter 4. We succeeded in the identification of chicken *SLC2A4* mRNA. We looked into databases (NCBI gene/nucleotide, Ensembl and Uniprot) and found only a partial sequence (XM_014262656) of glucose transporter 4 in *Pseudopodoces humilis*. Using this sequence we performed BLAST against the EST database to identify two partial fragments (EH289514.1 and EH286454.1) in turkey which are similar to mammalian glucose transporter 4. These sequences were the starting point for cloning of chicken *SLC2A4*. The partial sequences were cloned (using the AccuPrime polymerase) from chicken pectoralis muscle. This sequence was extended toward the 5’ end using RNA seq data (SRR924561 and SRR924559) and towards 3’ end using RNA seq data combined with 3’RACE. All predicted mRNA sequences were confirmed with PCR, followed by sequencing. Recently (17-06-2018), an update on the NCBI-server, revealed the identification of a sequence (XM_025145961; gene name still not correct: LOC107049937) of which the predicted protein is 503 amino acids long. Our identified sequence contains 519 amino acids. The difference in length is explained by the lack of the C-terminus in the online available sequence. The mRNA sequence encoding the C-terminus was the most difficult to obtain in our experimental setup (several rounds of nested PCR protocols combined with 3’RACE). The mRNA sequence of GLUT4 has a GC content of 69%,. The mRNA sequence can be found at the end of the file.

To ensure we have cloned GLUT4 we performed a pBLAST against human and the predicted protein sequence was most related to human GLUT4. The mRNA and protein identity with the human sequence was respectively 63 and 53%. The mRNA expression of *SLC2A4* in different tissues is shown in Fig. 5a, which is consistent with what is described in mammals: expression in adipose tissue and high expression skeletal muscle and heart.

| **Gene** | **ID** | **Species** | **Forward primers** | **Reverse primers** |
| --- | --- | --- | --- | --- |
| ALDOA | XM_005433403 XM_005534075 | *Falco cherrug Pseudopodoces humilis* | GTATCCTGGCTGCCGATGAGTCCAC | ACTCCTCCTGCGCCGCCTT |
|  |  |  | ATGGCCCCGCCCGT | TCAGTAGGCGTGGTTGGACA |
|  |  |  | CAGCATCTGCCAGCAGAACG | CGTTCTGCTGGCAGATGCTG |
| PYGM | XM_005533220 GGEZCB1010G06 GGEZCB1001H04 | *Pseudopodoces humilis Gallus gallus* | TGGACATGGAGGACCTGCAGGA | TCCTGCAGGTCCTCCATGTCCA |
|  |  |  | TCATCTACGAGATCAACCAGCGCTT | AAGCGCTGGTTGATCTCGTAGATGA |
|  |  |  | TACCACATGGCCAAGATGATCATCA | TGATGATCATCTTGGCCATGTGGTA |
|  |  |  | ACCTGTCGGAGCAGATCTCCA | TGGAGATCTGCTCCGACAGGT |
|  |  |  | AAGTCAGCGCCCTCTACAAGAAC | GTTCTTGTAGAGGGCGCTGACTT |
|  |  |  | ATGTCGCGACCGCTGASSGW | ATGGTGCCGGGGCTC |
|  |  |  | GTCAATATGCTCATGAACCACGAC |  |
|  |  |  | GTCAATATGCTCATGAACCACGAG |  |
| SLC2A4 (GLUT4) | XM_014262656 EH289514.1 EH286454.1 SRR924561 SRR924559 | *Pseudopodoces humilis Meleagris gallopavo Gallus gallus* | ATGCCGATTGGATTCCAGCAG | GGCGTTGGTGGCAATCATGG |
|  |  |  | ATGGCTGTGGGGACGGCA | CCGAGACCAGCCCTGAGTAGG |
|  |  |  | CTGGCCTTTGTGGGCGGGGCC | ACGACGGCCAATTGATGCAGG |
|  |  |  | GGGGCTCTGGGGACCCTGCACCA | AAGATCGCATTGATGCCGGAGAGCTGCTG |
|  |  |  | AGCTCTCCGGCATCAATGCGAT | GTGGCGGCCACGTTGACCAC |
|  |  |  | ACTCCACGGCCATCTTTGAAGG | GGCAGCGCTTCGTGGTCC |
|  |  |  | CGCTGCAGCACGCTCTG | GGCCACGCGTCGACTAGTACTTTTTTTTTTTTTTTTT |
|  |  |  |  | GGCCACGCGTCGACTAGTACT |

Sequence *ALDOA*

ATGCCTCACCAGTACCCAGCGCTCACCCCGGAGCAGAAGCAGGAGCTGCACGACATCGCCAAGCGCATCGTGGCGCCGGGCAAAGGCATCCTGGCTGCTGATGAGTCCACCGGCAGCATCGCCAAGCGGCTGAGCTCGGTGGGGGCGGAGAACACGGAGGAGAACCGGCGCTGGTACCGCCAGCTGCTGTTCACGGCCGACAACCGCGTGGATCCGTGCATCGGCGGAGTCATCCTCTTCCATGAGACCCTCTACCAGAAGGCCGACGACGGCCGCCCCTTCCCTCAGGTCATCAAGGCCAAGGGAGGGGTCGTCGGGATCAAGGTTGACAAAGGCGTTGTGCCCCTGGCTGGCACCAATGGGGAGACCACCACCCAGGGGCTGGACGGGCTGATGGAGCGCTGCGCTCAGTACAAGAAGGACGGGGCTGACTTTGCCAAGTGGCGCTGCGTGCTGAAGATCTCGGAGCACACCCCGTCCCGCCTGGCCATCATGGAGAACGCCAACGTCCTGGCACGATACGCCAGCATCTGCCAGCAGAACGGCATTGTCCCCATCGTGGAGCCCGAAATCCTCCCTGACGGTGACCACGACCTCAAACGCTGCCAATACGTCACTGAGAAGGTCCTGGCAGCGGTGTACAAAGCCCTGAGCGACCACCACATTTACCTGGAGGGCACTTTGCTCAAACCCAACATGGTGACGGCGGGCCATTCGTGCACCAAGAAGTACAGCCCCGAAGAGATCGCCATGGCCACCGTCACCGCCCTGCGCCGCACCGTCCCCCCCGCAGTGCCTGGCATCACCTTCCTCTCCGGAGGCCAAAGTGAGGAGGAGGCCTCGGTCAACCTGAACGCCATCAACCGCTGCCCGCTTCACCGGCCGTGGGCCCTCACCTTCTCCTACGGGCGGGCGCTGCAGGCCTCGGCCCTCAAGGCCTGGAGCGGCAAGAAGGAGAACACCAAGGCGGCCCAGGAGGAGTACGTCAAGAGGGCCTTGGCCAACTCCCTGGCATGCCAAGGGAAATACACCCCCAGCGGCCACGCGGGGGCGGCCGCCAGCGAATCCCTCTTCATGTCCAACCACGCCTACTGA

Sequence *PYGM*

ATGTCGCGACCGCTGAGGGACCACGAGAAGCGGAAGCAGATCAGCGTGAGGGGCCTCGCGGGCGCCGAGAACGTCTCGGAGCTGAAGAAGGGCTTCAATCGGCACCTGCACTTCACGTTGGTCAAAGACCGCAATGTGGCCACCAGCCGCGATTACTACATGGCCCTGGCGCACACCGTGCGCGACCACCTGGTGGGCCGATGGCTGCGCACGCAGCAGCACTACTACGAGAAGGACCCCAAGCGCATCTATTATCTGTCGTTGGAGTTCTACATGGGCCGCACTCTGCAGAACACAATGATCAACCTCGGGCTGCAGAGCTCCTGCGACGAGGCCGTGTATCAGCTGGGCCTGGACATGGAGGACCTGCAGGAGATCGAGGAGGACGCCGGGCTCGGCAATGGCGGCCTGGGGCGCCTGGCAGCGTGCTTCCTGGACTCCATGGCCACGCTGGGTTTGGCTGCTTAYGGTTACGGCATCCGCTATGAGTTCGGCATCTTCAACCAGAAGATCGCGGGCGGATGGCAGGTGGAGGAGGCGGACGATTGGCTGCGCTATGGGAACCCGTGGGAAAAGGCGCGTCCCGAATACACCATTCCGGTGCACTTCTATGGGCGGGTGGAGCACTCCCACGACGGGGCCAAGTGGTTGGACACACAGGTGGTGCTGGCGCTGCCGTACGACACTCCGGTGCCGGGTTACCGCAACAACACAGTGAACACCATGCGGCTGTGGTCCGCCCGCGCCCCCAACGACTTCAACCTCAAAGACTTCAATGTGGGCGGTTACATTCAGGCGGTGTTGGATCGCAATTTGGCTGAGAACATTTCCCGCGTGCTCTACCCCAACGACAACTTCTTTGAGGGGAAGGAGCTGCGTTTGAAGCAGGAGTACTTTGTGGTGGCGGCGACGCTGCACGATGTGGTGCGGCGCTTCAAATCCGCCAAATTCGGCAGCCGCGACCCCGTGAGGACGCACTTCGACTCCTTCCCCGATAAGGTGGCCATCCAGCTGAACGACACGCACCCCTCTCTCGCCATCCCGGAGCTGATGCGCATTCTGGTGGATGAGGAGAAGCTCGGCTGGGATAAGGCGTGGGACATCACGGTGCGGACGTGCGCCTACACGAACCACACGGTGCTGCCGGAGGCGTTGGAGCGTTGGCCCGTGGGGCTGCTGGAGGCGCTGCTGCCGCGGCACCTCGAGATCATCTACGAGATCAACCGCCGCTTCCTCGACCGCGTGTACGCGTCGTTCCCGGGGGACCACGACCGCCTGCGCCGGATGTCGTTGGTGGAGGAGGGGGCGGTGAAGCGCATCAATATGGCGCACCTCTGCATCGCCGGCGCGCACGCGGTCAACGGCGTCGCGCAGATCCACTCCGACATCCTCAAGCACAGCGTCTTTAAGGATTTCTATGAGTTGGACCCTCAGAAGTTCCAGAACAAAACCAATGGGATCACTCCGCGCCGATGGCTGCTGCTCTGCAACCCGGGGCTGGCAGAGGTCATCGCCGAGCGCATCGGGGATGAGTTTGTGGCCGACCTGGATCAGCTGCAGCGCCTGAAGGCGTTGGTGGACGACGACGCCTTCATCCGCGACGTGGCCAAAGTGAAGCAGGAGAACAAGGCGAAGTTCGCGGCGCAGTTGGAGCGCGATTATGGGCTGCGCGTGAACCCGGCGGGGCTGTTTGACGTGCAGGTGAAGAGGATCCACGAATACAAACGGCAGCTGCTCAACTGCCTGCACGTCATCACCCTCTACAACAGGATTAAGAAGGAACCGAATAAACCCTTCGTGCCCCGAACCGTTATGATTGGTGGAAAGGCGGCCCCCGGGTACCACATGGCGAAGCTGATCATCAAACTGATCACATCCATCGGCGATGTGGTGAACCACGACCCGGCCGTGGGCGACCGCCTGAAGGTCATCTTCCTCGAGAACTACCGCGTGTCGCTGGCCGAGAAGGTGATCCCGGCCGCAGACCTATCGGAGCAGATCTCGACGGCCGGCACGGAGGCGTCGGGGACGGGCAACATGAAGTTCATGCTGAACGGGGCGCTGACCATCGGCACCATGGACGGCGCCAACGTGGAGATGGCGGAGGAGGCGGGAGAGGAAAACCTCTTCATCTTCGGCATGAGGGTGGAGGACGTGGAGCGCCTCGACCGCCAGGGTTACTGTGCCCGCGACTACTACGAGCGGCTGCCGGAGCTGCGCACGGCGGTGGATCAGCTGAGCAGCGGTTTCTTCAGCCCGCGGCAGCCCGATCTGTTCCGGGACATCGTCAATATGCTCATGAACCACGACCGATTTAAGGTTTTCGCTGACTACGAATCGTACGTCAAATGCCAGGAGCGCGTCAGTGAGCTCTACAAGGACTCGCGGCAGTGGACGCGGACGGTGATTCGGAACATCGCGGCGGCGGGGAAATTCTCGAGCGACCGAACCATCGCGCAGTACGCGCGCGAGATTTGGGGCACCGAACCCACCCGGCACCGCATCCCCGCGCCCGACGAGCCCCGGCACTGA

Sequence *SLC2A4* (GLUT4)

ATGCCGATTGGATTCCAGCAGATCCAGAATGAGGAGGAGGACGCGGATGCGCCGCCCCCCCCCGGGCTGACCCCCACCCTGGCCATGGCGGTGGGGACGGCGGTTTTGGGGTCACTGCAGGTCGGATACCACGTGGGGGTCATCAACGCACCCCAAAAGGTCCTGGAAGATGAATACAACACGACGTGGACGCAGCGCTGGGGGGAGCCGCCCCCCCCCACGACCGTCTCCACCCTCTGGGCTCTGTCGGTCGCCATCTTCTCCGTGGGGGGGATGATGGCATCGCTGATGGTGGGCGTGGTGGCTGAGCGCCTCGGCAGGAAGCACGCCATGATTGCCACCAACGCCTTGGCCTTTGTGGGCGGGGCCATGATGGGCGCGGCCAAGTGGGGCCCCTCCTACGTCCTCATCATCATTGGTCGGTTCCTGCTCGGCGCCTACTCAGGGCTGGTCTCGGGGCTGGTGCCAATGTACGTGGGGGAGATCGCCCCCACCCACCTACGGGGGGCTCTGGGGACCCTGCATCAATTGGCCGTCGTCATCGGCATCCTGGGGGCACAGGTGCTGGGCCTGGGAGCCCTGCTGGGGACGGCGCGGCGCTGGCCGCTGCTGCTGGGATTGGGGCTGTGCCCGGCGGCCCTGCAGGCTCTGCTGCTGCCGCGCTGCCCTGAGAGCCCCCGATTCCTCCTGGGGAGAGAGCGCCGTGGGGCTGCCATGCATGCCCTCACGCTCCTATTGGGTCCGGAGGCGGCGGAGGCGGAGCTTGAGGCGCTGGGGGCGGAGCTTCGAGGCCCCACCCCCCGAATGGGGGTGCTCCGCCTCCTGGGGAGCCCCCGGCTGCGACAGCCATTGGTGGTGGCCATTGGGCTACAGCTCAGCCAGCAGCTCTCCGGCATCAATGCGATCTTCTATTACTCCACGGCCATCTTTGAAGGCGCGGGGGTGGGGCAGCCCGCCGTGGCCACCATCGGGGTCGGGGTGGTCAACGTGGCCGCCACGTTGCTCTCGCTGTTCCTGGTGGAGCGCTCCGGCCGCCGCACTCTGCAGCTGGTGGGGATCGGGGGGATGTTGGTCTGTGCCATCTGCCTCACCGCCAGCCTGCGCCTGCAGGACTCCCCGGGCGCGGGCGCTGTCAGCCTGCTGGGGGTTTTCCTCTTTGTCACCTTCTTTGAGTTGGGCCCCGGTCCCATCCCGTGGTTCCTGGCGGCAGAGCTCTTCCCTCAGGGACCCCGACCTGCAGCCGTGGCCTTGGCTGGAGCTGCCAATTGGGCCGGGAACTTCGTGGTGGGGATGGCCTTCCCTGCGCTGCAGCACGCTCTGGGCCCGCTGGTATTCGTGCTGTTCGCCGCCCTGTTGGGAGCTTCGTGGCTCTTCGCTTTCTATCTGCTGCCCGAAACCAGAGGGAGACCCTTCGAGGGGGGGGACCACGAAGCGCTGCCCCCCCCCCACCACCACCACCCCCCCCACGGGACCCCCCCGCACCCATTGCTGGGAGGGGGGGGGGGAGAGGTGAAGGGGGGCACTGAACTGCAGCCGCTGAGGGGGGGGGAAGATGGCTGA

References

1. Schettino CM, Lima DF, Leyton JF, El-Dorry HA, Bacila M. Studies on the structure of aldolase A from chicken muscle. Biochim. Biophys. Acta. 1981;667:411–20.

2. Russell GA, Dunbar B, Fothergill-Gilmore LA. The complete amino acid sequence of chicken skeletal-muscle enolase. Biochem. J. 1986;236:115–26.

3. Tanaka M, Maeda K, Nakashima K. Chicken α-enolase but not β-enolase has a src-dependent tyrosine-phosphorylation site: cDNA cloning and nucleotide sequence analysis. J. Biochem. 1995;117:554–9.

4. Sokolove PM. Altered membrane association of glycogen phosphorylase in the dystrophic chicken. Biochim. Biophys. Acta. 1985;841:232–6.
